# Assemblies of JAG1 and JAG2 determine tracheobronchial cell fate in mucosecretory lung disease

**DOI:** 10.1172/jci.insight.157380

**Published:** 2022-08-08

**Authors:** Susan D. Reynolds, Cynthia L. Hill, Alfahdah Alsudayri, Scott W. Lallier, Saranga Wijeratne, Zheng Hong Tan, Tendy Chiang, Estelle Cormet-Boyaka

**Affiliations:** 1 Center for Perinatal Research,; 2 Institute for Genomic Medicine, and; 3 Center for Regenerative Medicine, Nationwide Children’s Hospital, Columbus, Ohio, USA.; 4 School of Veterinary Medicine, The Ohio State University, Columbus, Ohio, USA.

**Keywords:** Cell Biology, Stem cells, Adult stem cells, Asthma, COPD

## Abstract

Mucosecretory lung disease compromises airway epithelial function and is characterized by goblet cell hyperplasia and ciliated cell hypoplasia. Goblet and ciliated cell types are derived from tracheobronchial stem/progenitor cells via a Notch-dependent mechanism. Although specific arrays of Notch receptors regulate cell fate determination, the function of the ligands Jagged1 (JAG1) and JAG2 is unclear. This study examined JAG1 and JAG2 function using human air-liquid-interface cultures that were treated with γ-secretase complex (GSC) inhibitors, neutralizing peptides/antibodies, or WNT/β-catenin pathway antagonists/agonists. These experiments revealed that JAG1 and JAG2 regulated cell fate determination in the tracheobronchial epithelium; however, their roles did not adhere to simple necessity and sufficiency rules. Biochemical studies indicated that JAG1 and JAG2 underwent posttranslational modifications that resulted in generation of a JAG1 C-terminal peptide and regulated the abundance of full-length JAG2 on the cell surface. GSC and glycogen synthase kinase 3 were implicated in these posttranslational events, but WNT agonist/antagonist studies and RNA-Seq indicated a WNT-independent mechanism. Collectively, these data suggest that posttranslational modifications create distinct assemblies of JAG1 and JAG2, which regulate Notch signal strength and determine the fate of tracheobronchial stem/progenitor cells.

## Graphical abstract

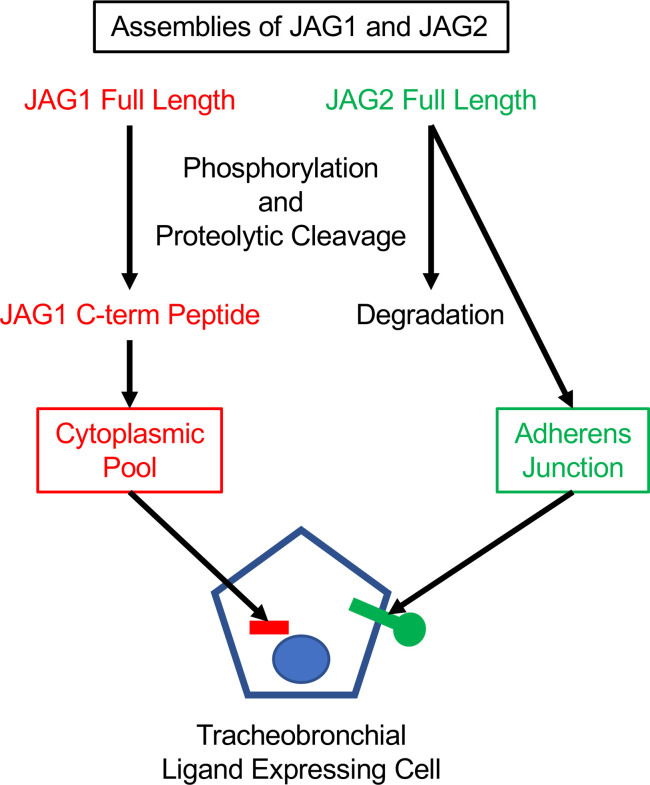

## Introduction

The conducting airway epithelium protects the lung from inspired environmental agents via mucociliary clearance. Effective removal of pathogens, irritants, and toxins (including cigarette smoke and air pollutants) requires the coordinated activity of two specialized airway epithelial cell types, the multiciliated cell (termed a ciliated cell) and secretory cells (including goblet cells and club cells). These cell types are descendants of the tracheobronchial epithelial tissue–specific stem cell (TSC), which regenerates the pseudostratified airway epithelium and resides in the trachea and bronchi of the mouse and upper respiratory tract of humans. Lineage tracing in mice (1–4) and clonal analysis in human (5) has indicated that the TSC was a basal cell subtype.

Previous studies have reported that tracheobronchial ciliated and secretory cells were present in approximately equal numbers and that their frequency was regulated by Notch signaling (reviewed in refs. 6, 7). According to this model, signaling occurred between adjacent cells, which expressed Notch ligands (jagged1 [JAG1] or JAG2 or delta-like 1 [DLL1], DLL3, or DLL4) or notch receptors (NOTCH1, -2, -3, or -4) (6, 8). Following receptor ligation, ADAM10 or ADAM17 cleaved NOTCH at an extracellular site and the γ-secretase complex (GSC) cleaved NOTCH at an intracellular site. This process released the NOTCH intracellular domain, which formed a transcriptional complex with several proteins, including RBPJ and activated expression of the HES and HEY family of transcriptional repressors (9–11).

Chronic lung disease is frequently associated a disruption of mucociliary clearance. In asthma, chronic obstructive pulmonary disease, chronic bronchitis, and idiopathic pulmonary fibrosis, goblet cell hyperplasia and excess mucus secretion drive the mucosecretory phenotype. Single-cell RNA-Seq (scRNA-Seq) analysis of asthma, idiopathic pulmonary fibrosis, and chronic obstructive pulmonary disease indicated that Notch signaling was aberrant (12–14), and functional studies indicated that cigarette smoke, an agent that increases risk of developing chronic obstructive pulmonary disease, chronic bronchitis, or idiopathic pulmonary fibrosis, activated Notch signaling (15). Mucociliary clearance can also be disrupted by ozone and cigarette smoke exposure or infection with SARS-CoV-2 and/or respiratory syncytial virus. These agents damage the ciliated cell, often resulting in cell death and ciliated cell hypoplasia. Notch pathway components were identified as susceptibility genes for lung damage (16), viral entry (17), and hyperinflammation (18). Consequently, treatments that specifically target Notch pathway components have the potential to normalize ciliated and goblet cell frequency, restore mucociliary clearance, and improve the health of people with chronic lung disease.

Several studies defined the Notch receptors responsible for generation of ciliated and secretory cells in mouse and human (13, 19–23). However, the Notch ligand(s) that was responsible for pathway activity was unclear. Studies in mice indicated that DLL ligands regulated generation of neuroepithelial cells but were unlikely to be involved in production of ciliated and secretory cells (24). In contrast, overexpression of *Jag1* or *Jag2* (21, 25) and colocalization of the 2 mRNAs (24, 26) suggested overlapping roles for JAG1 and JAG2 in basal cell fate determination. Conditional knockout of *Jag1* or *Jag2* early in mouse lung development supported this overlapping function, but other studies identified JAG2 as the dominant ligand (24, 26). Finally, a specific role for JAG1 in maintenance of secretory cell fate and suppression of ciliated cell fate was indicated by experiments involving *JAG1* overexpression in immortalized human basal cells (27), analysis of *Jag1*-knockout mice (28), and treatment of mice with a JAG1-neutralizing antibody (29) or a Jag1 antisense oligonucleotide (30).

A potential explanation for the disparate Jag results was the focus on the fully differentiated ciliated or goblet cell. Ciliated cell subtypes were identified by scRNA-Seq (31–33), while histological analysis of the mouse tracheobronchial epithelium (34) and the Xenopus epidermis (reviewed in ref. 35) defined ciliated cell differentiation intermediates. These intermediates can be detected by immunostaining for acetylated tubulin (ACT; ref. 36). Similarly, multiple goblet cell subtypes have been identified and vary according to the amount and subcellular location of MUC5B (36–38).

This study examined JAG1 and JAG2 regulation of tracheobronchial cell fate determination using human bronchial air-liquid-interface (ALI) cultures. This widely utilized model of the tracheobronchial epithelium (39) was optimized to allow detection of changes in cell type frequency following treatment with neutralizing peptides/antibodies, GSC inhibitors (GSIs), and WNT/β-catenin pathway antagonists/agonists. Biochemical approaches were used to examine JAG1 and JAG2 secretion, trafficking, and posttranslational processing.

## Results

### Differentiation model development.

Analysis of JAG function required a model system in which changes in cell frequency could be detected. Consequently, we evaluated ciliated and goblet cell differentiation in 3 media that are commonly used during the differentiation phase of ALI culture (Supplemental Methods and Supplemental Figures 1–3; supplemental material available online with this article; https://doi.org/10.1172/jci.insight.157380DS1). This study showed that Half&Half (H&H) medium supported an increase in ciliated and goblet cell frequency between day 7 and day 14 and generated similar numbers of ciliated and goblet cells on day 14; it also showed that the cells expressed furin-activated NOTCH1, -2, and -3. Based on these findings, the H&H model was used to evaluate the signaling mechanisms that regulate production of ciliated and goblet cells.

### Kinetics of ciliated and goblet cell differentiation.

**Figure 1:**
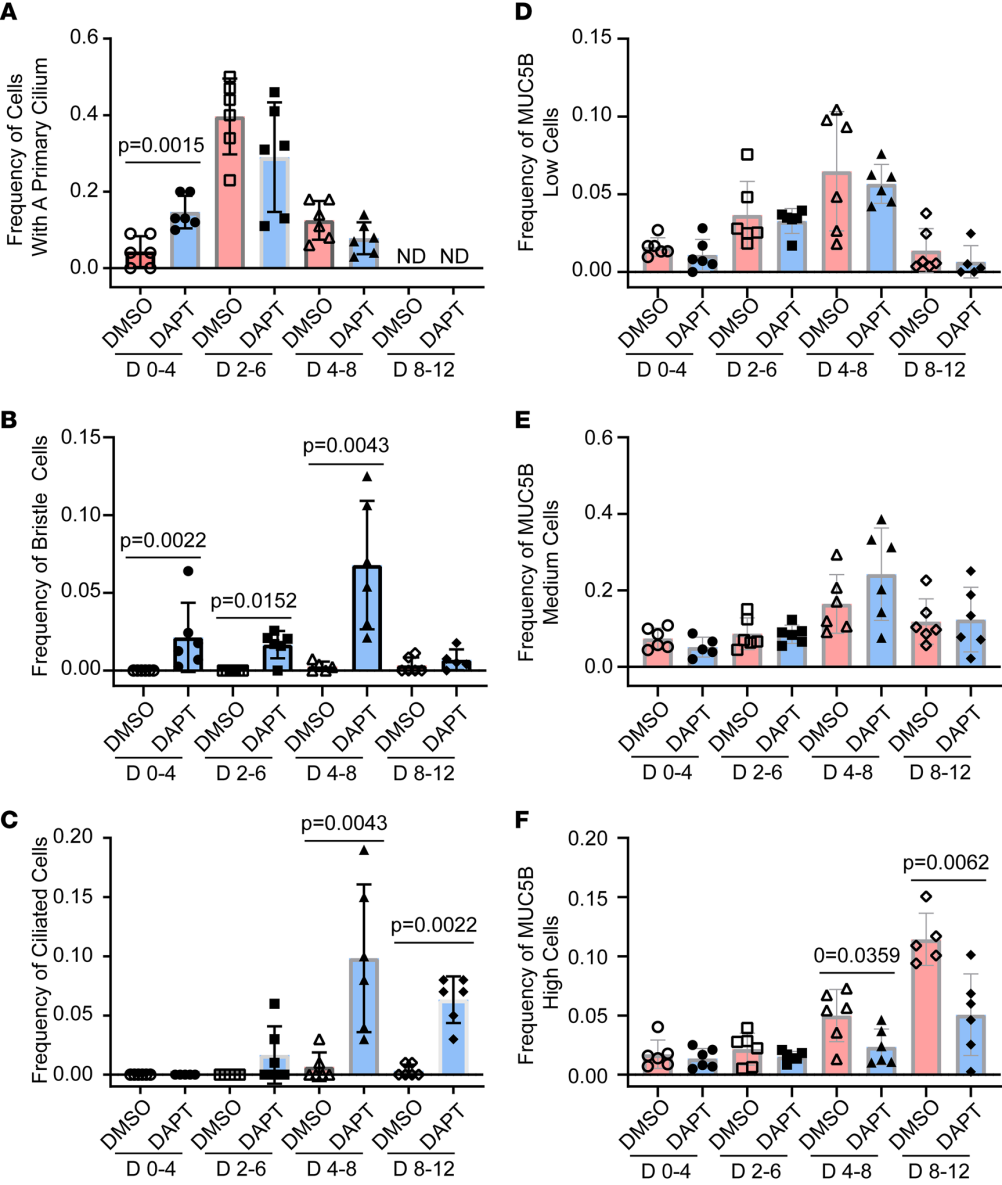
Notch regulation of ciliated and goblet cell differentiation. Human bronchial basal cells were differentiated in ALI cultures using H&H medium. Cells were treated with vehicle (DMSO) or 25 μM DAPT as follows: treatment on day 0 and day 2 and fixation on day 4, treatment on day 2 and day 4 and fixation on day 6, treatment on day 4 and day 6 and fixation on day 8, or treatment on day 8 and day 10 and fixation on day 12. Dual immunofluorescence was used to detect ACT and MUC5B. Nuclei were stained with DAPI. (**A**) Frequency of cells with a primary cilium. (**B**) Frequency of bristle cells. (**C**) Frequency of ciliated cells. (**D**) Frequency of MUC5B-low cells. (**E**) Frequency of MUC5B-medium cells. (**F**) Frequency of MUC5B-high cells. All quantitative data are presented as the mean ± SD, *n* = 6. Normally distributed data were analyzed by *t* test. Nonnormally distributed data were analyzed by Mann-Whitney test.

Ciliated and goblet cell differentiation intermediates were identified by immunostaining for ACT and MUC5B (Supplemental Methods and Supplemental Figure 2). Cells with a primary cilium were detected at early time points, and the frequency of these cells peaked on differentiation day 6 (Figure 1A). Bristle cells were identified on days 8–12 (Figure 1B), and mature ciliated cells were detected on days 8–12 (Figure 1C). All 3 goblet cell differentiation intermediates were detected at all time points (Figure 1, D–F). However, the frequency of MUC5B-low and MUC5B-medium cells peaked on day 8, while the frequency of MUC5B-high cells increased through day 12 (Figure 1F). These data indicated that the first wave of ciliated and goblet cell differentiation occurred between day 0 and day 12.

### Notch regulation of ciliated and goblet cell differentiation.

To determine if Notch regulated differentiation, cultures were treated with N-[N-(3,5-difluorophenacetyl)-_L_-alanyl]-S-phenylglycine t-butyl ester (DAPT), the GSI that was used to evaluate Notch signaling in the mouse tracheobronchial epithelium (26). DAPT treatment on days 0–4, days 2–6, days 4–8, and days 8–12 did not alter cell density (Supplemental Figure 3E). Compared with treatment with control, DAPT treatment significantly increased the frequency of cells with a primary cilium on day 4 (Figure 1A), significantly increased the frequency of bristle cells on days 4, 6, and 8 (Figure 1B), and significantly increased the frequency of ciliated cells on day 8 and day 12 (Figure 1C). DAPT treatment did not alter the frequency of MUC5B-low or MUC5B-medium cells at any time point (Figure 1, D and E). However, DAPT treatment significantly decreased the frequency of MUC5B-high cells on day 8 and day 12 (Figure 1F). These data complemented previous analysis of Notch signaling during terminal differentiation by showing that Notch regulated early ciliated and goblet cell fate determination.

### Expression of JAG1 and JAG2 in ALI cultures.

Dual immunofluorescence was used to determine if ALI cultures expressed JAG1 or JAG2. On day 7, all cells were JAG1 positive, and the protein localized to the perinuclear/nuclear compartments (Supplemental Figure 5). JAG2 was also detected (Supplemental Figure 5) and was highly enriched in the cortical domain. JAG2 signal intensity in the cortical region varied from dim to bright.

### Roles for JAG1 and JAG2 in Notch signaling.

A previous study reported that a JAG1-DSL domain peptide antagonized Notch signaling (40); however, other reports showed that this reagent agonized Notch signaling (41, 42). Because the JAG1 peptide is 71% identical to the JAG2-DSL domain, inconsistent results could be due to loss of JAG1 and/or JAG2 function. To address this controversy, 3 experiments were conducted.

**Figure 2:**
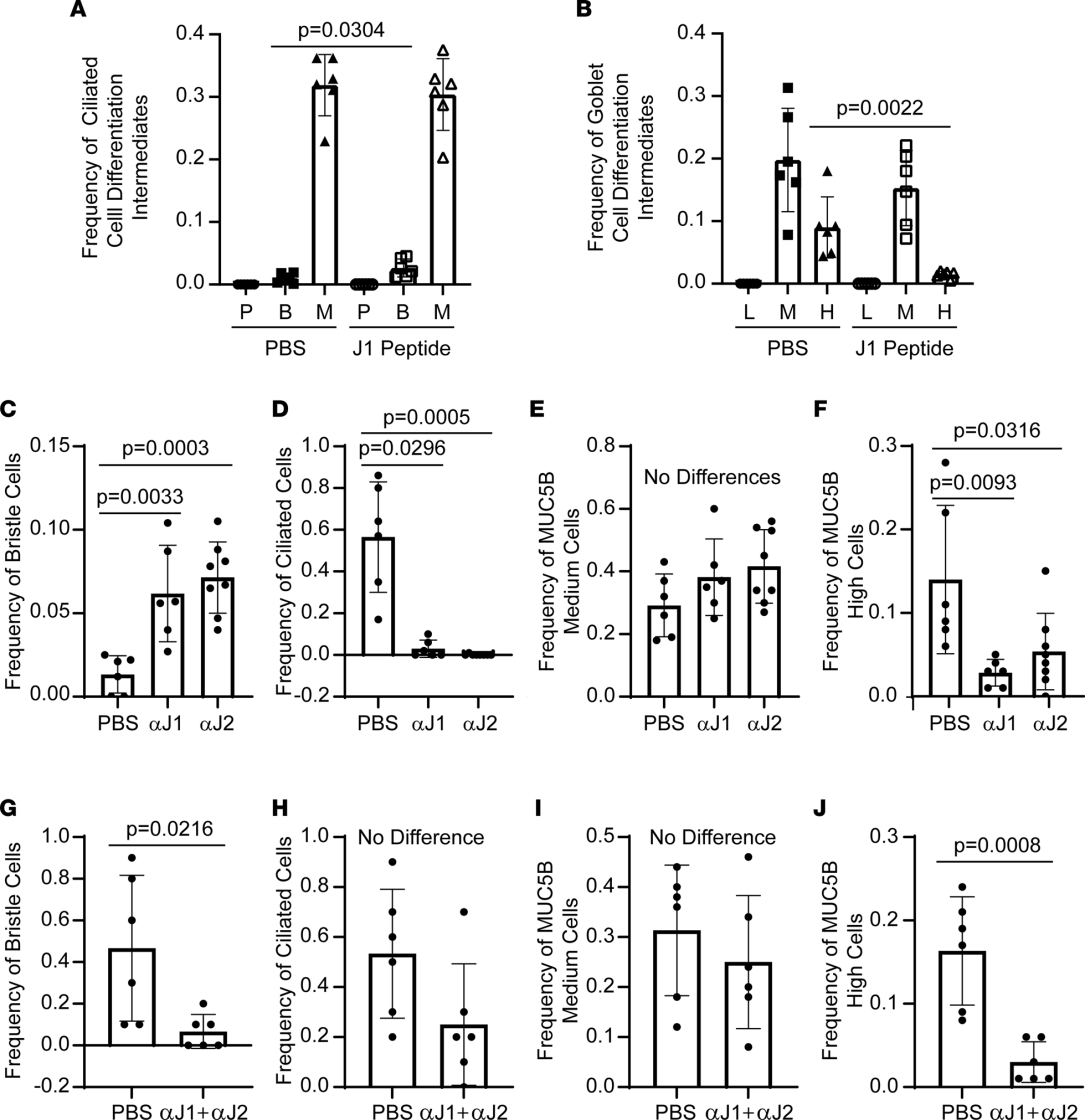
Roles for JAG1 and JAG2 in ciliated and goblet cell differentiation. Human bronchial basal cells were differentiated in ALI cultures using H&H medium. Treatments were on day 8 and day 10, and cultures were fixed on day 12. Differentiation was quantified by immunofluorescence analysis of ACT, MUC5B, and DAPI. (**A** and **B**) Cells were treated with vehicle (PBS) or 40 μM JAG1 peptide. (**A**) Frequency of ciliated cell differentiation intermediates. P, cells with a primary cilium, B, bristle cells; M, ciliated cells. (**B**) Frequency of goblet cell differentiation intermediates. L, MUC5B-low cells; M, MUC5B-medium cells; H, MUC5B-high cells. (**C–F**) Cells were treated with vehicle (PBS) or 25 μg/mL neutralizing antibody against JAG1 (αJ1) or JAG2 (αJ2). (**C**) Frequency of bristle cells. (**D**) Frequency of ciliated cells. (**E**) Frequency of MUC5B-medium cells. (**F**) Frequency of MUC5B-high cells. (**G–J**) Cells were treated with PBS or 25 μg/mL αJ1 and 25 μg/mL αJ2. (**G**) Frequency of bristle cells. (**H**) Frequency of ciliated cells. (**I**) Frequency of MUC5B-medium cells. (**J**) Frequency of MUC5B-high cells. All quantitative data are presented as the mean ± SD, *n* = 6. Normally distributed data were analyzed by *t* test. Nonnormally distributed data were analyzed by Mann-Whitney test.

First, ALI cultures were treated with the JAG1-DSL domain peptide on day 8 and day 10 and cultures were fixed on day 12. Immunofluorescence analysis of ACT and MUC5B was used to detect differentiation intermediates. Cells expressing a primary cilium were not detected (Figure 2A). However, vehicle-treated cultures contained a small number of bristle cells and many ciliated cells. Treatment with the JAG1 peptide caused a significant increase in the frequency of bristle cells but did not alter the frequency of ciliated cells (Figure 2A). The same cultures did not contain MUC5B-low cells (Figure 2B). However, MUC5B-medium and MUC5B-high cells were detected. Treatment with JAG1 peptide did not alter the frequency of MUC5B-medium cells but significantly decreased the frequency of MUC5B-high cells.

Second, ALI cultures were treated with JAG1- or JAG2-specific neutralizing antibodies (29) on day 8 and day 10 and fixed on day 12. Like the JAG1-DSL peptide study, treatment with anti-JAG1 or anti-JAG2 significantly increased bristle cell frequency (Figure 2C) and decreased the frequency MUC5B-high cells (Figure 2F). However, neutralization of JAG1 or JAG2 also decreased ciliated cell frequency (Figure 2D). Neutralization of JAG1 or JAG2 did not alter MUC5B-medium cell frequency (Figure 2E).

Finally, ALI cultures were treated with a mixture of the anti-JAG1 and anti-JAG2 antibodies. This treatment significantly decreased bristle cell frequency (Figure 2G) but did not alter ciliated cell frequency (Figure 2H). Furthermore, the combined neutralizing antibody treatment did not alter the frequency of MUC5B-medium cells (Figure 2I) and significantly decreased the frequency of MUC5B-high cells (Figure 2J). Collectively, these studies indicated that JAG1 and JAG2 were involved in tracheobronchial cell fate determination. However, the finding that JAG1 and JAG2 function did not adhere to simple necessity and sufficiency rules suggested a complex regulatory mechanism. Because ligand trafficking and posttranslational modifications are known to regulate Notch signaling (43), these processes were investigated in the ALI model.

### JAG1 subcellular location and molecular weight.

**Figure 3:**
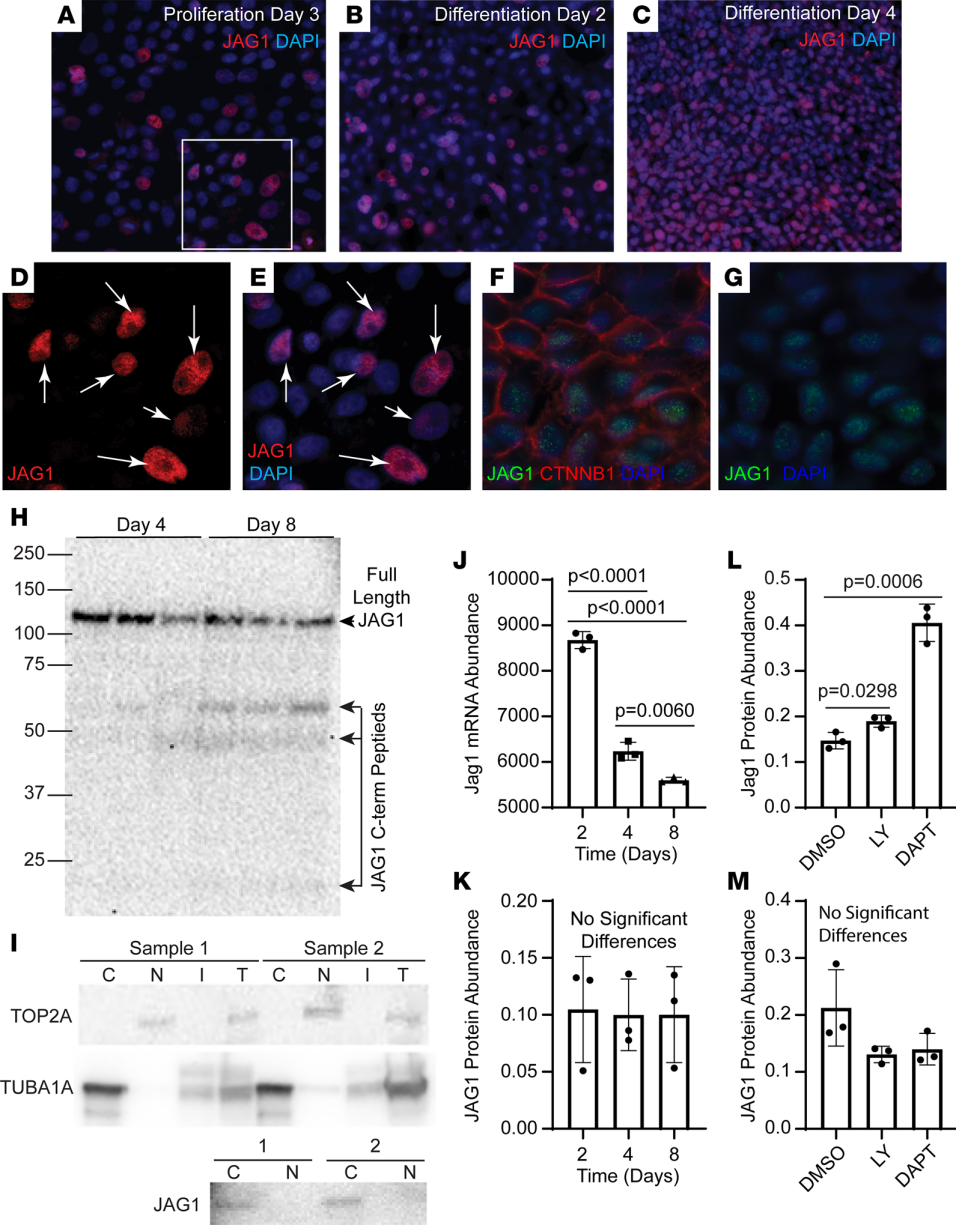
JAG1 subcellular location. Human bronchial basal cells were differentiated in ALI cultures using H&H medium. (**A–C**) Immunofluorescence was used to detect JAG1 (red) on proliferation (**A**) day 3, (**B**) day 2, or (**C**) day 4. Nuclei were detected with DAPI (blue). Original magnification, ×400. (**D** and **E**) High-magnification images of the boxed region in **A**. Arrows indicate JAG1-positive cells. Original magnification, ×800. (**F** and **G**) Dual immunofluorescence analysis of JAG1 (green) and CTNNB1 (red) on day 4. Nuclei were detected with DAPI (blue). (**F**) Three-color and (**G**) 2-color images of the same region. Original magnification, ×800. (**H**) Western blot analysis of JAG1 on day 4 and 8. The full-length band is labeled JAG1. C-terminal fragments are shown by the bracket. Three samples per time point. (**I**) Cells were cultured to day 8, separated into cytoplasmic (C), nuclear (N), and insoluble (I) fractions or were unfractionated (T), and proteins were analyzed by Western blot. Top: TOP2A, a nuclear protein; middle: TUBA1A, a cytoplasmic protein; bottom: analysis of full-length JAG1 in the C and N fractions. Two different samples were analyzed. (**J**) Analysis of *Jag1* mRNA abundance on day 2, day 4, and day 8. Mean ± SD (*n* = 3). (**K**) Western blot analysis of full-length JAG1 protein abundance on day 2, day 4, and day 8. Mean ± SD (*n* = 3). (**L** and **M**) Western blot analysis of full-length JAG1 abundance in cultures that were (**L**) treated with DMSO, 25 μM DAPT, or 10 μM LY on day 4 and day 6 and harvested on day 8 or (**M**) treated on day 8 and day 10 and harvested on day 12. All quantitative data are presented as the mean ± SD, *n* = 3. Normally distributed data were analyzed by *t* test. Nonnormally distributed data were analyzed by Mann-Whitney test.

Initial studies showed that JAG1 was concentrated in a perinuclear or nuclear domain on day 7 (Supplemental Figure 5). To determine when this pattern was established, JAG1 distribution was evaluated at earlier time points. On proliferation day 3 (Figure 3, A, D, and E) and day 2 (Figure 3B), JAG1 was expressed in a subset of cells and was in the perinuclear/nuclear domain. JAG1 did not colocalize with β-catenin (CTNNB1), a component of the adherens junction (AJ; Figure 3, F and G). On day 4 (Figure 3C), JAG1 was detected in most cells, and some variation in protein abundance was noted.

Previous studies reported that Notch ligands can be cleaved by the GSC, resulting in creation of a transcriptionally active 25 kDa C-terminal fragment (44–46). Consequently, JAG1 molecular weight was determined using a JAG1 antibody that detected the C-terminal domain. On day 4 and day 8, all samples contained full-length (~130 KDa) JAG1, and day 8 samples contained faint C-terminal fragments (Figure 3H). To further examine JAG1 subcellular localization, cytoplasmic and nuclear fractions were isolated on day 8. Detection of DNA topoisomerase II α (TOP2A) in the nuclear fraction and α-tubulin (TUBA1A) in the cytoplasmic fraction (Figure 3I) demonstrated successful separation. Full-length JAG1 was limited to the cytoplasmic fraction, which indicated that the protein was perinuclear rather than nuclear.

Because the previous studies indicated that most if not all JAG1 was in a perinuclear compartment and that JAG1 might be processed posttranslationally, the relationship between transcription and translation was evaluated. *Jag1* mRNA was quantified by RNA-Seq, and full-length JAG1 protein levels were quantified by Western blot. *Jag1* abundance decreased as a function of time (Figure 3J). In contrast, JAG1 abundance was constant over time (Figure 3K). To determine if GSI treatment altered JAG1 abundance, cultures were treated with vehicle, DAPT, or LY450139 (LY; a functionally distinct GSI) on day 4 and day 6, and protein expression was analyzed by Western blot on day 8. GSI treatment increased the abundance of full-length JAG1 (Figure 3L and Supplemental Figure 4D). In contrast, GSI treatment on day 8 and day 10 did not alter the abundance of full-length JAG1 on day 12 (Figure 3M and Supplemental Figure 4E). These data suggested that JAG1 was processed by the GSC at early time points and raised the possibility that JAG1 function was regulated posttranscriptionally.

### JAG2 subcellular location and molecular weight.

**Figure 4:**
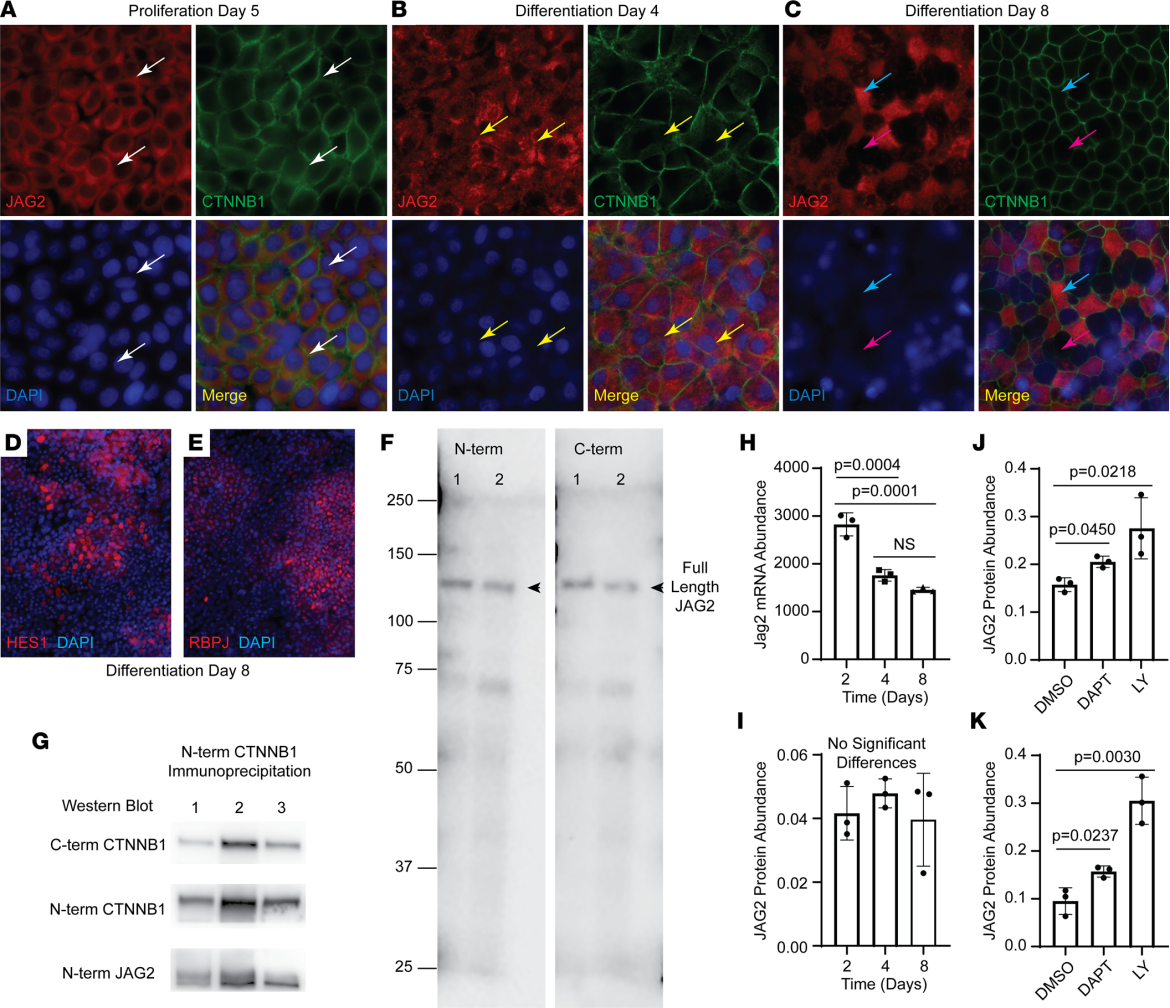
JAG2 subcellular location. Human bronchial basal cells were differentiated in ALI using H&H medium. (**A–C**) Immunofluorescence was used to detect JAG2 (red) and CTNNB1 (green) on proliferation (**A**) day 5, (**B**) day 4, or (**C**) day 8. Nuclei were detected with DAPI (blue). Each montage presents single-color and merged images. White arrows, cortical JAG2; yellow arrows, JAG2-high cells; blue arrows, JAG2-positive cells; pink arrows, JAG2-negative cells. Original magnification, ×400. (**D** and **E**) Immunofluorescence analysis of (**D**) HES1 (red) and (**E**) RBPJ (red) on day 8. Nuclei were detected with DAPI (blue). Original magnification, ×200. (**F**) Western blot analysis of JAG2 on day 4 (lane 1) and day 8 (lane 2). The full-length band is labeled JAG2. Two samples were analyzed. (**G**) Coimmunoprecipitation analysis of full-length JAG2 and CTNNB1 on day 8. Proteins were immunoprecipitated with an N-terminus–specific (N-term–specific) CTNNB1 antibody. Precipitates were analyzed for CTNNB1 using a C-terminus–specific (C-term–specific) CTNNB1 antibody, N-term–specific CTNNB1 antibody, or a JAG2 N-term–specific antibody. Three samples were analyzed. (**H**) Analysis of *Jag2* mRNA abundance on day 2, day 4, and day 8. (**I**) Western blot analysis of full-length JAG2 protein abundance on day 2, day 4, and day 8. (**J** and **K**) Western blot analysis of full-length JAG2 abundance in cultures that were (**J**) treated with DMSO, 25 μM DAPT, or 10 μM LY on day 4 and day 6 and harvested on day 8 or (**K**) treated on day 8 and day 10 and harvested on day 12. All quantitative data are presented as the mean ± SD, *n* = 3. Normally distributed data were analyzed by *t* test. Nonnormally distributed data were analyzed by Mann-Whitney test.

A preliminary analysis suggested that JAG2 expression-level varied among cells on day 7 (Supplemental Figure 5). Consequently, JAG2 localization was further evaluated on proliferation day 5, day 4, and day 8 (Figure 4, A–C). The cortical pattern was observed on proliferation day 5 and JAG2-high cells were apparent on day 4. By day 8, JAG2-positive and -negative cells were noted. At each time point, JAG2 colocalized with CTNNB1. On day 8, groups of cells expressing nuclear HES1 (Figure 4D) and RBPJ were detected (Figure 4E) and indicated focal Notch signaling.

The molecular weight of JAG2 was determined using 2 antibodies that were specific to the N-terminal or C-terminal domains. This study detected full-length (~145 KDa) JAG2 on day 4 and day 8 (Figure 4F). To further examine JAG2 subcellular localization, coimmunoprecipitation was used to determine if JAG2 was associated with the AJ. An N-terminal specific CTNNB1 antibody was used for immunoprecipitation, and the precipitate was analyzed with N-terminal and C-terminal specific CTNNB1 antibodies. CTNNB1 was successfully isolated (Figure 4G), and JAG2 was identified in the CTNNB1 complex.

Because the previous studies suggested that JAG2 abundance varied, the relationship between transcription and translation was evaluated using RNA-Seq and Western blots. *Jag2* abundance decreased as a function of time (Figure 4H). In contrast, JAG2 abundance was constant over time (Figure 4I). To determine if GSI treatment altered JAG2 abundance, cultures were treated with vehicle, DAPT, or LY, as indicated above. GSI treatment increased the abundance of full-length JAG2 on day 8 and day 12 (Figure 4, J and K, and Supplemental Figure 4, F and G). These data suggested that JAG2 was continuously processed by the GSC and raised the possibility that JAG2 function was regulated posttranscriptionally.

### Abundance of JAG1 and JAG2 at the cell surface.

**Figure 5:**
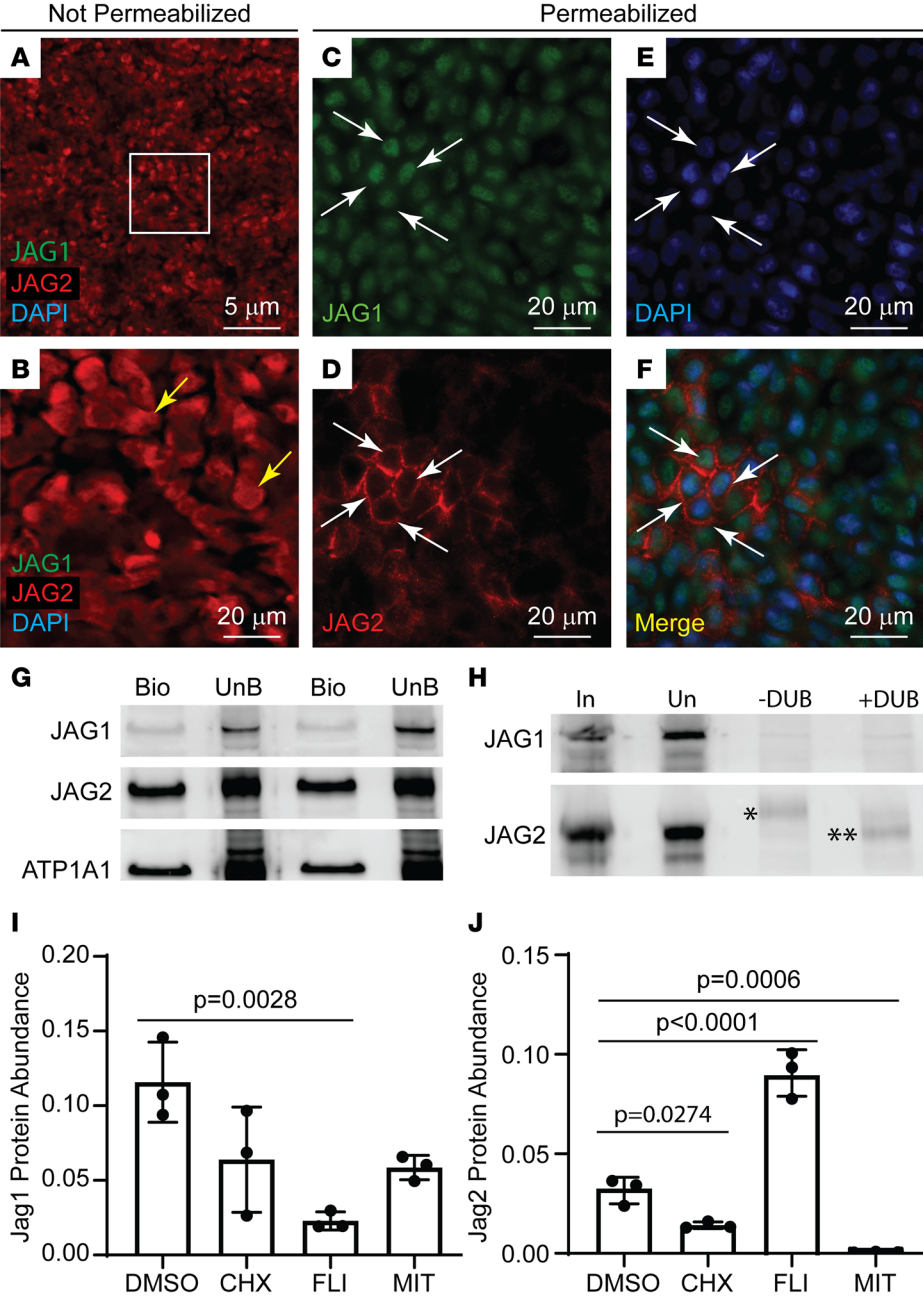
JAG1 and JAG2 trafficking to the cell surface. Human bronchial basal cells were differentiated in ALI cultures using H&H medium. (**A** and **B**) On day 8, cultures were fixed under nonpermeabilization conditions, and JAG1 (green) and JAG2 (red) were detected by dual immunofluorescence staining. Nuclei were stained with DAPI (blue). (**B**) A higher-magnification image of the boxed region in **A**. Yellow arrows, JAG2-positive cells. Scale bars: 5 μm (**A**); 20 μm (**B**). (**C–F**) After imaging, the cultures were permeabilized and restained for JAG1 (green), JAG2 (red), and DAPI (blue). Arrows, antigen-positive cells. Scale bars: 20 μm. (**G**) On day 8, cell surface proteins were labeled with biotin, biotinylated proteins were recovered, and the bound (BIO) and unbound (UnB) fractions were evaluated by Western blot. Two samples were evaluated for full-length JAG1, JAG2, or ATP1A1. (**H**) On day 8, cell lysates were reacted with TUBE beads that bind to proteins decorated with 4 or more ubiquitin moieties. The bound fraction was treated with vehicle (-DUB) or a broad spectrum debubiquitinase (+DUB). Western blots were generated from the input (In), unbound (Un), -DUB, and +DUB samples and were evaluated for full-length JAG1 and JAG2. *, Ubiquitinated JAG2; **, deubiquitinated JAG2. (**I** and **J**) On day 7, cultures were treated with vehicle (DMSO), 1.77 μg/mL cycloheximide (CHX), 10 μM FLI-06 (FLI), or 10 μM MITmAB (MIT) and lysed for Western blot analysis of (**I**) full-length JAG1 and (**J**) full-length JAG2. All quantitative data are presented as the mean ± SD, *n* = 3. Normally distributed data were analyzed by *t* test. Nonnormally distributed data were analyzed by Mann-Whitney test.

To further investigate roles for JAG1 and JAG2 in Notch signaling, cell surface protein was evaluated by immunofluorescence analysis of nonpermeabilized cultures. JAG1 was not detected on the cell surface on day 8. In contrast, JAG2 was detected on the surface of many cells (Figure 5, A and B). Subsequent permeabilization and staining detected both JAG1 and JAG2 and illustrated their typical intracellular distributions (Figure 5, C–F).

Cell surface biotinylation was used to evaluate JAG1 and JAG2 trafficking to the plasma membrane. These studies detected sodium potassium ATPase-α1 (ATP1A1), a known cell surface protein, as well as JAG1 and JAG2 (Figure 5G). However, cell surface JAG2 was 5–10 times more abundant than JAG1.

Because JAG1 and JAG2 are polyubiquitinated and internalized after interaction with a Notch receptor, this process was evaluated using Tandem Ubiquitin Binding Entity (TUBE) technology. This approach detects adducts containing 4 or more ubiquitin units. JAG1 was not detected in the bound (ubiquitin-positive) fraction (Figure 5H). In contrast, JAG2 was detected in the bound fraction, and the molecular weight of the captured JAG2 decreased after treatment with a deubiquitinase (Figure 5H). These biochemical studies indicated that most JAG1 was cytoplasmic, whereas most of JAG2 was located at the AJ.

### JAG1 and JAG2 secretion and recycling.

The literature indicates that ligand trafficking during secretion (protein movement from the endoplasmic reticulum [ER] to the PM) and recycling (protein movement from the PM to the AJ) determine the ability of the ligand to activate a Notch receptor (47). Whether this process involves posttranslational modifications and/or enrichment of ligands in specialized membrane domains is an area of active investigation (48).

To evaluate JAG1 and JAG2 secretion, day 7 ALI cultures were treated with 1.77 μg/mL cycloheximide (protein synthesis inhibitor) for 24 hours. Cycloheximide treatment did not alter JAG1 abundance but resulted in an approximately 50% decrease in JAG2 abundance (Figure 5, I and J). These data suggested that the cell contained a stable reservoir of JAG1 and that the half-life of JAG2 was approximately 24 hours.

Next, 10 μM FLI-06 was used to inhibit protein movement from the ER to the Golgi (49). FLI-06 decreased JAG1 (Figure 5I), which suggested that movement from the intermediate compartment to the Golgi (50–52) generated an intracellular JAG1 reservoir. In contrast, FLI-06 treatment increased JAG2 abundance (Figure 5J). These data suggested that JAG2 was secreted via a Golgi-independent route and that this process protected JAG2 from degradation.

To evaluate JAG1 and JAG2 recycling, ALI cultures were treated with 10 μM MiTMAB, a dynamin/endocytosis inhibitor. This treatment did not alter JAG1 abundance (Figure 5I) and supported the idea that most JAG1 was intracellular. In contrast, MiTMAB treatment significantly decreased JAG2 abundance (Figure 5J), indicating that JAG2 endocytosis and recycling to the AJ protected JAG2 from degradation. Collectively, the analysis of JAG1 and JAG2 location, ubiquitination, and trafficking supported the idea that JAG1 functioned intracellularly and JAG2 operated at the cell surface.

### Regulation of JAG1 and JAG2 abundance.

**Figure 6:**
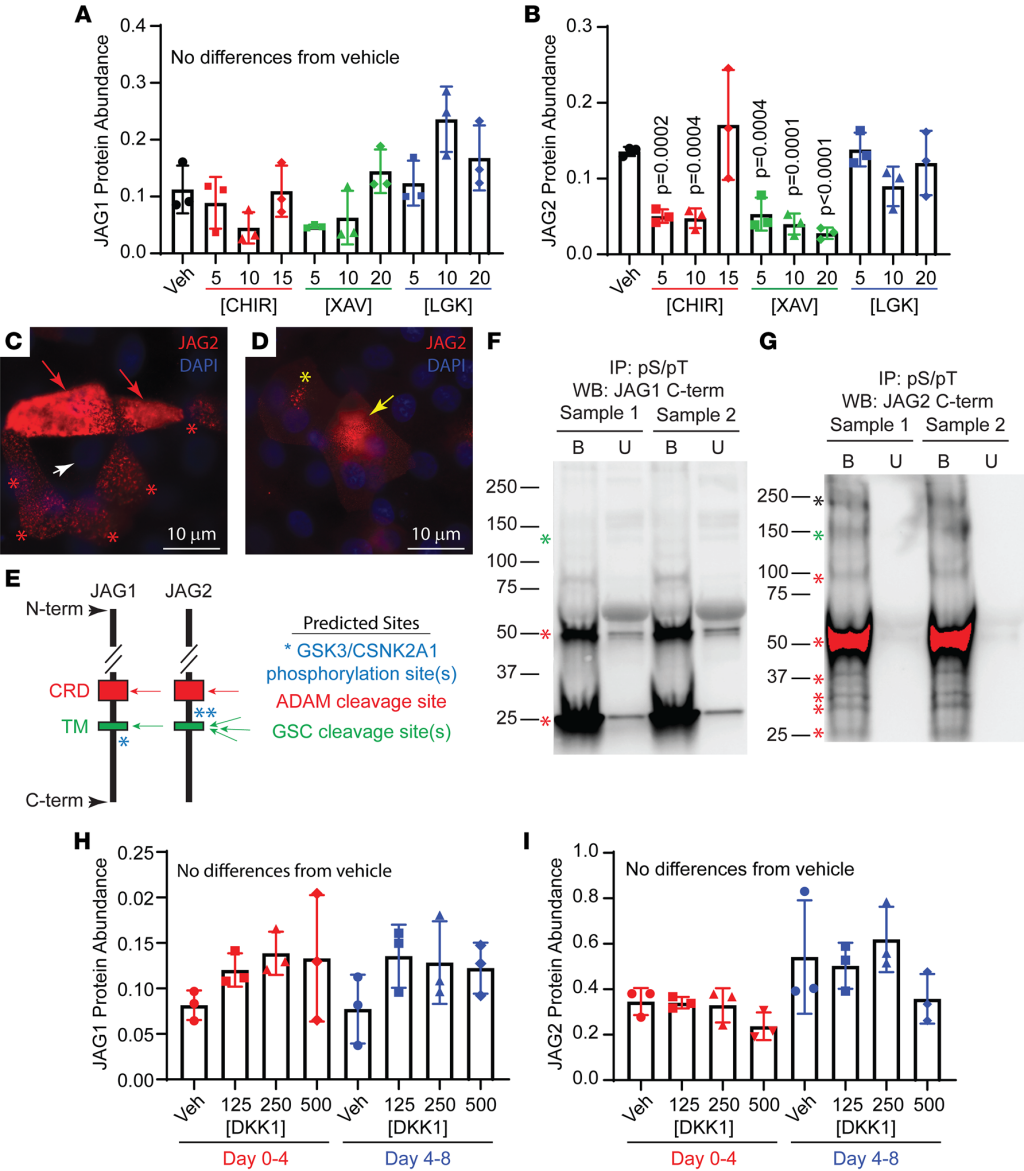
Regulation of JAG1 and JAG2 abundance. Human bronchial basal cells were differentiated in ALI cultures using H&H medium. (**A** and **B**) On day 4 and day 6, the cultures were treated with vehicle (Veh; DMSO), CHIR99021 (CHIR), XAV939 (XAV), or LGK974 (LGK), and protein lysates were collected on day 8. CHIR and XAV concentrations are in μM. LGK concentrations are in pM. Western blots were used to (**A**) quantify full-length JAG1 and (**B**) full-length JAG2 abundance. Mean ± SD, *n* = 3. (**C** and **D**) Immunofluorescence was used to detect JAG2 in (**C**) Veh- and (**D**) CHIR-treated (5 μM) cultures on day 8. Red arrows, JAG2-high cell; white arrow, JAG2-negative cell; yellow arrow, redistributed JAG2; asterisks, JAG2-low cells. Scale bars: 10 μm. (**E**) JAG1 and JAG2 primary structure is represented by the vertical line with the amino-terminus (N-term) at the top and the carboxy-terminus (C-term) at the bottom. The red boxes represent the cysteine-rich domain (CRD), and green boxes represent the transmembrane domain (TM). Drawings are not to scale. Putative GSK3/CSNK phosphorylation sites are represented by asterisks, ADAM is represented by red arrows, and GSC cleavage sites are represented by green arrows. (**F** and **G**) Cells were cultured to day 8, lysed, and immunoprecipitated with a phospho-serine/phospho-threonine (pSer/pThr) antibody. Precipitates were analyzed for (**F**) JAG1 and (**G**) JAG2 using C-terminus specific antibodies. Two samples were analyzed. Black asterisk, high-molecular-weight protein; green asterisks, full-length protein; red asterisks, C-terminal fragments. (**H** and **I**) Cultures were treated with Veh or DKK1 on day 0 and day 2 and lysed on day 4 or treated on day 4 and day 6 and lysed on day 8. Drug concentrations are in ng/mL. Western blots were used to quantify (**H**) full-length JAG1 and (**I**) full-length JAG2 abundance. All quantitative data are presented as the mean ± SD, *n* = 3. Normally distributed data were analyzed by *t* test. Nonnormally distributed data were analyzed by Mann-Whitney test.

The GSI studies suggested that JAG1 and JAG2 were GSC targets but did not explain the presence of JAG2-positive and JAG2-negative cells on day 8. Because previous studies suggested that the WNT/β-catenin pathway regulated Notch signaling (36), ALI cultures were treated with various concentrations of CHIR99021 (CHIR; a WNT/β-catenin agonist, ref. 53) or XAV939 (XAV; a WNT/β-catenin antagonist, ref. 54) on day 4 and day 6, and cell lysates were collected on day 8. Western blot analysis demonstrated that neither CHIR nor XAV treatment altered the abundance of JAG1 (Figure 6A and Supplemental Figure 4H). In contrast, both treatments caused a significant decrease in JAG2 abundance (Figure 6B and Supplemental Figure 4H). Interestingly, the CHIR effect was limited to lower doses (5 and 10 μM), whereas the XAV effect was observed at all 3 doses. Western blot analysis of total CTNNB1, active CTNNB1, glycogen synthase kinase 3 (GSK3), phospho-serine GSK3, and AXIN demonstrated efficacy of the CHIR and XAV treatments (Supplemental Figure 4, H–M).

### Phosphorylation of JAG1 and JAG2.

Immunofluorescence analysis demonstrated that CHIR treatment caused a striking redistribution of JAG2 to a perinuclear location (Figure 6, C and D) and was suggestive of JAG2 degradation. JAG2 was not detected by immunofluorescence staining in XAV treated cells (data not shown). CHIR is a potent and highly selective inhibitor of GSK3 (53). In contrast, XAV inhibits tankyrase and regulates GSK3 by altering the kinase’s localization within the cell (54). Since both drugs decrease GSK3 activity and JAG2 abundance, it was possible that the shared mechanism was a GSK3-dependent decrease in JAG2 phosphorylation.

Based on reports that GSK3 phosphorylates targets that are primed by casein kinase 1 (CSNK1A1) or CSNK1A2 (55, 56) and that JAG proteins were putative GSK3/CSNK targets (57), NetPhos-3.1 was used to scan JAG1 and JAG2 for GSK3 (S/TXXXS/T) and CK (S/TXXE/D) sites. This analysis showed that JAG1 contains a GSK3/CSNK consensus site on the C-terminal side of the transmembrane domain (aa1101-SHTHSASEDNT-aa1111, Figure 6E) and that JAG2 contains a double GSK3/CSNK site on the N-terminal side of the transmembrane domain (aa1015-DRASSGASAVE-aa1025, Figure 6E). In addition to different locations, these sites had distinct sequences (0% identity and 18%–34% conservation).

To determine if JAG1 and/or JAG2 were phosphorylated, day 8 cell lysates were immunoprecipitated with an anti–phosphorylated-serine/phosphorylated-threonine (pS/pT), antibody, and Western blots were probed for JAG1 and JAG2 (Figure 6, F and G). The majority of pS/pT-JAG1 was processed to 50 and 25 KDa fragments that contained the C-terminal domain (Figure 6F). The sharpness of these bands suggested two well-defined cleavage sites. In contrast with JAG1, pS/pT-JAG2 exhibited a complex pattern that included high-molecular-weight and full-length bands, an abundant 60 kDa C-terminal fragment, and 4 bands in the 25–39 kDa range (Figure 6G). Collectively, these data suggested that JAG1 and JAG2 were by phosphorylated by GSK3 and this posttranslational processing might be coupled with site-specific proteolytic cleavage by ADAM10/17 proteases and the GSC (Figure 6E).

### WNT-independent regulation of JAG1 and JAG2 abundance.

To examine the effect of WNT antagonism on JAG1 and JAG2 abundance, ALI cultures were treated with vehicle; LGK974, a porcupine inhibitor that prevents WNT secretion; or DKK1, which inhibits WNT signaling by binding the LRP5/6 coreceptor. Neither LGK974 treatment (Figure 6, A and B, and Supplemental Figure 4H) nor DKK1 treatment (Figure 6, H and I) altered JAG1 or JAG2 abundance. These data suggested that a WNT-independent mechanism regulated the abundance of JAG2 in CHIR- and XAV-treated cultures.

### Effect of CHIR and XAV on the transcriptome.

CHIR and XAV are typically thought to regulate the WNT/β-catenin pathway by altering gene expression. To evaluate this mechanism, ALI cultures were treated with vehicle, 10 μM CHIR, or 10 μM XAV on day 4 and day 6, and RNA was purified on day 8. RNA-Seq and Gene Set Expression Analysis (GSEA) demonstrated that CHIR upregulated expression of WNT/β-catenin target genes, including *Axin2*, *Lef1*, and *Tcf7* (Supplemental Figure 6, A and B, and Supplemental Figure 7). These genes contributed to the GSEA leading edge gene set and had a high degree of effect on the GSEA enrichment score (an indicator of changes in pathway activity). Other GSEA leading edge genes included *Wnt5B* and known pathway antagonists (*Axin1, Dkk1*, *Dkk4,* and *Nkd1*). Because the antagonists and *Axin2* inhibit WNT stabilization of CTNNB1 and *Lef1* and *Tcf7* repress gene expression in the absence of CTNNB1 (58), these data suggested that CHIR treatment antagonized the WNT/β-catenin pathway on day 8.

In keeping with its perceived function as a WNT/β-catenin pathway antagonist, XAV treatment downregulated expression of *Axin2*, *Lef1*, and *Tcf7* as well as *Wnt5B* (Supplemental Figure 6, C and D, and Supplemental Figure 7). However, most GSEA leading edge WNT/β-catenin pathway genes were typical of the Notch pathway (*Dll1*, *Hey1*, *Jag1*, *Jag2*, *Notch1*, and *Notch4*). Analysis of the Notch gene set indicated that XAV treatment caused a significant downregulation of Notch signaling (Supplemental Figure 6, G and H). A similar analysis of the Notch gene set in CHIR-treated cultures also indicated downregulation (Supplemental Figure 6, E and F). Although a high FDR *q* value suggested cautious interpretation of the CHIR/Notch pathway interaction, many of the GSEA leading edge genes were shared between the CHIR and XAV groups (*Dll1*, *Jag1*, *Notch1*, *Lfng*, *Prkca*, *Psenen*, *Sap30*, and St3gal6; Supplemental Figure 5, E–H, and Supplemental Figure 7).

*Jag1* and *Jag2* contributed to the enrichment scores; however, neither gene product was consistently included in the GSEA leading edge gene sets. Consequently, the effect of CHIR and XAV treatment on gene expression was evaluated. *Jag1* and *Jag2* abundance did not vary by treatment (Supplemental Figure 6, I and J). Collectively, the gene expression data indicated that the main effect of CHIR or XAV treatment was to antagonize WNT/β-catenin and Notch signaling on day 8 and that decreased signaling could not be attributed to a change in *Jag1* or *Jag2* gene expression.

### CHIR regulation of cell fate.

**Figure 7:**
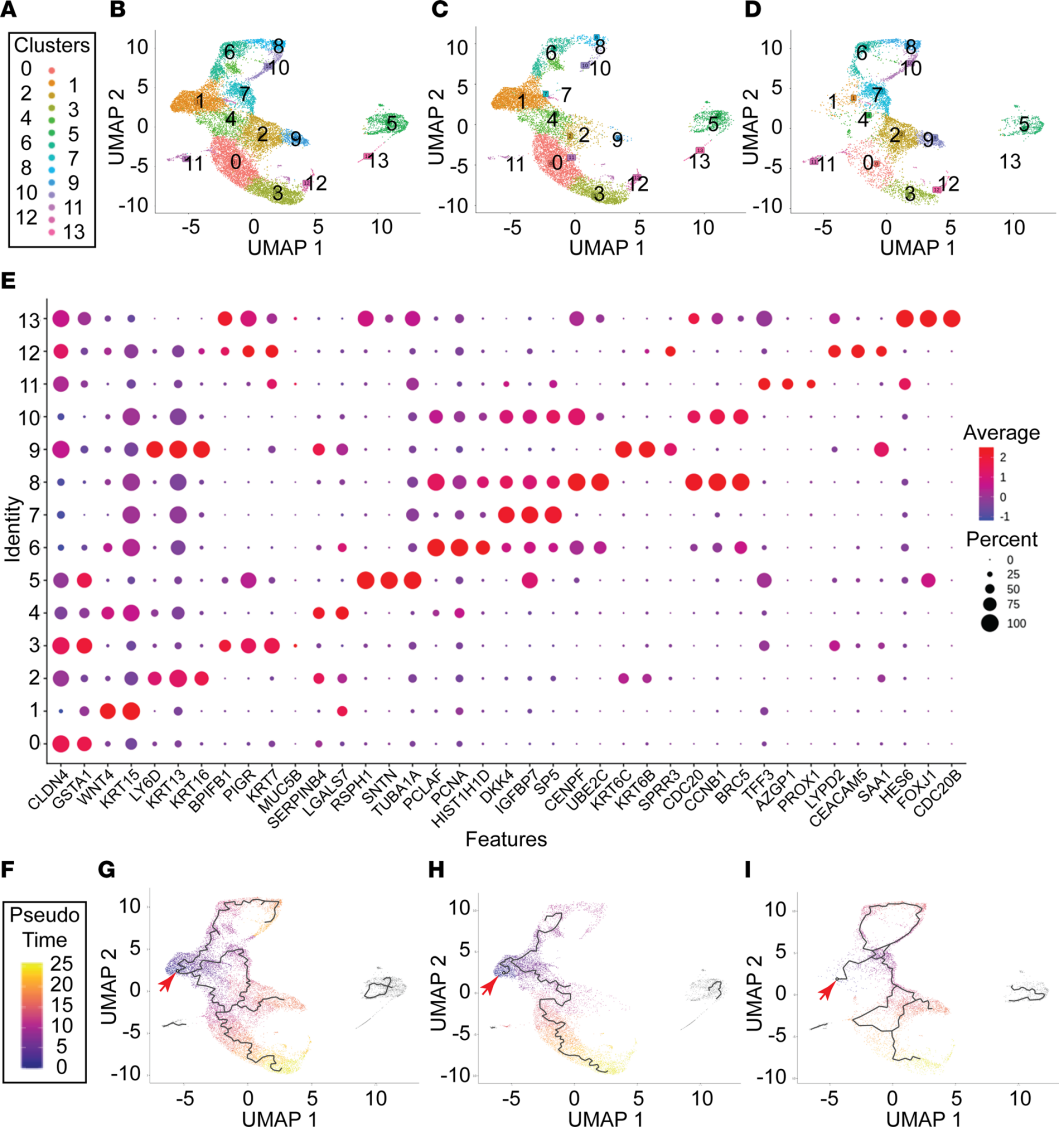
scRNA-Seq analysis of human bronchial basal cell differentiation. Human bronchial basal cells were differentiated in ALI cultures using H&H medium, treated with vehicle (DMSO) or 10 μM CHIR on day 4 and day 6, and harvested for scRNA-Seq analysis on day 8. (**A–D**) Uniform Manifold Approximation and Projection (UMAP) analysis. (**A**) Cluster identification color code. UMAP analysis of cells that were treated with (**B**) vehicle or CHIR, (**C**) vehicle, or (**D**) CHIR. (**E**) Dot plot of the marker genes used to identify the clusters. For each dot, the color gradient (blue to red) indicates the mean marker expression, and the size indicates the percentage of cells expressing the marker. See Supplemental Table 1 for the complete gene list. Cluster identification by cell phenotype or state: 0, secretory lineage, presecretory primed; 1, classical basal cells; 2, differentiating basal cells; 3, secretory lineage, secretory primed cell; 4, secretory lineage, presecretory primed; 5, ciliated lineage, multiciliated cells; 6, self-renewing cells; 7, motile/squamous cells; 8, self-renewing cells; 9, motile/squamous cells; 10, differentiating basal cells; 11, ciliated lineage; 12, secretory lineage, mature secretory cells; 13, ciliated lineage, bristle/deuterostome cells. (**F–I**) Inference of basal, secretory, squamous, and ciliated cell lineages by Monocle 3. (**F**) Pseudotime is represented by the purple (start) to yellow (end) gradient and is (**G**) based on an aggregate of the entire experiment, (**H**) vehicle-treated and (**I**) CHIR-treated cells. The red arrows indicate the assigned root node, and the differentiation trajectory is indicated by the black line.

To further examine changes in cell fate, ALI cultures were treated with CHIR on day 4 and day 6, and cells were isolated for scRNA-Seq in day 8. Fourteen clusters (clusters 0–13) were identified (Figure 7, A–E). Treatment resulted in marked differences in the frequency of cells in 10 of the clusters (Figure 7, B–E, and Supplemental Figure 8C). In vehicle-treated controls, clusters 6, 8, and 10 exhibited a mitotic signature (Supplemental Table 1). The frequency of cells in these clusters increased in CHIR-treated cultures. Cluster 1 was prominent in vehicle-treated cultures and contained basal cells that exhibited a classical signature (Figure 7E). This cluster overexpressed *Jag2* and *Wnt4* (Supplemental Table 1 and Supplemental Figure 8, A and B) and was nearly absent in CHIR-treated cultures. Clusters 0, 3, 4, and 12 contained cells within the secretory lineage (Figure 7E and Supplemental Table 1) and overexpressed *Hes4* (Supplemental Table 1 and Supplemental Figure 8, A and B). Cluster 3 contained secretory primed basal cells that were defined by expression of *Ceacam6*, *Scgb1A1*, and *Scgb3A2* (Figure 7E and Supplemental Table 1). Cells within cluster 3 were less mature than those in cluster 12. Clusters 0, 4, and 3 were markedly decreased in CHIR-treated cultures (Supplemental Figure 8C). Clusters 5, 11, and 13 contained ciliated lineage cells. Custer 13 contained bristle cells, while cluster 5 contained mature ciliated cells. Clusters 11 and 13 were defined by overexpression of the Notch-independent gene *Hes6* (59) (Figure 7E, Supplemental Table 1, and Supplemental Figure 8, A and B). CHIR treatment increased the frequency of cluster 11 cells and decreased the frequency of cluster 13 cells (Supplemental Figure 8C), which suggested a progenitor progeny relationship. Clusters 5 and 13 contained cells that expressed the Notch targets *Hes1*, *Hes2*, and *Hes4* (Supplemental Figure 8, A, B, and D). Cluster 2 was increased in CHIR-treated cultures, and clusters 7 and 9 were unique to CHIR-treated cultures (Supplemental Figure 8C). Cluster 7 was distinguished by expression of cell motility and keratinization signatures (Figure 7E and Supplemental Table 1), and this keratinization signature was upregulated in clusters 2 and 9. Because keratinization was previously associated with squamous differentiation in the tracheobronchial epithelium (60), these and previous data sets indicated that CHIR treatment promoted the squamous fate and suggested that JAG2 suppressed squamous differentiation.

To integrate the scRNA-Seq study with our analysis of secretory cell differentiation intermediates (ref. 36, Figure 1, and Supplemental Figure 2) and reports of a CEACAM6-positive secretory primed basal cell (13), we used pseudotime analysis to predict cell lineage relationships (ref. 61 and Figure 7, F–I). Analysis of vehicle-treated cells indicated that classical basal cells (cluster 1) generated a self-renewing population (clusters 6, 8, and 10) and a CEACAM6^low/–^ population (cluster 4) (Figure 7H). The trajectory split at this level and gave rise to rare squamous cells (cluster 2) and a second CEACAM6^lo/–^ cell type that expressed some goblet/club cell markers (cluster 0). The first CEACAM6-positive populations (clusters 2 and 3) generated mature goblet and club cells (cluster 12). Very few immature ciliated cells (cluster 13) were detected, and this may explain the lack of a secretory to ciliated cell trajectory.

To further investigate squamous differentiation, pseudotime analysis was used to examine cell trajectories in CHIR-treated cultures (Figure 7I). Consistent with the idea that CHIR induced squamous metaplasia, this study found that treatment enhanced differentiation of cluster 4 cells to the squamous fate (clusters 2 and 9). Collectively, these data positioned the MUC5B-low cell in cluster 2 and suggested that it was a progenitor for the secretory primed basal cell and most likely the progenitor for squamous cells (clusters 2 and 9).

## Discussion

Previous studies indicated that tracheobronchial cell fate was determined by different assemblies of Notch receptors (13, 20). The present study suggests that different assemblies of JAG ligands may determine Notch signal intensity. First, JAG1 and JAG2 regulated cell fate within the secretory and ciliated lineages, but their function could not be defined by a simple set of rules. Second, JAG1 was detected in all differentiating cells. A small amount of full-length JAG1 was located on the cell surface, but little of this protein was trafficked to the AJ. In contrast, most JAG1 was a 25 kDa C-terminal peptide that was restricted to a perinuclear domain. Third, a striking change in cell morphology coincided with the emergence of JAG2-positive and -negative cells and raised the possibility that increased polarization concentrated JAG2 at the AJ (62). Within the JAG2-positive cell subset, the amount of cell surface JAG2 was also regulated, potentially by the GSC. When combined with the histological and biochemical data, these studies indicated that Notch signal intensity was regulated by the amount of full-length JAG2 at the AJ and refined by the intracellular pool of cleaved JAG1. This model, if supported by further analysis, it may explain inconsistencies in the *Jag1*/JAG1 and *Jag2*/JAG2 literature.

The present study also indicated that progression through the ciliated cell differentiation process was regulated by a GSC-sensitive target and raised the possibility that Notch signaling might transition from “off” to “on” as the cell progressed from one morphological intermediate to the next. Previous studies reported that ciliated cell differentiation was regulated by an oscillator, potentially the ciliated transcription network gene product, *cMyb*. The abundance of this gene product fluctuated over time and regulated progression through the ciliated cell differentiation program (63). Although this transcriptional mechanism was supported by expression of Notch targets in ciliated lineage cells, demonstration that JAG1 was processed to a nonnuclear C-terminal domain peptide suggested that JAG1 might regulate ciliated cell differentiation via an alternative mechanism.

Previous studies showed that transmembrane proteins, including JAG1 and JAG2, transit the endocytic recycling compartment as they move to the plasma membrane. This compartment is positioned under the nucleus in adherent cells and sorts endocytosed proteins that are recycled to the plasma membrane, enter the lysosome for degradation, or are sequestered in a perinuclear site termed the pericentrion (64). A protein’s trafficking pattern is determined in part by phosphorylation and ubiquitination and changes in the rate of movement through the various endosomal compartments regulate Notch signaling (65).

The present study identified a striking difference in the subcellular location of JAG1 and JAG2. Cell fractionation, biotinylation, and ubiquitination studies indicated that JAG1 was highly enriched in the perinuclear domain, and secretion, trafficking, and phosphorylation studies indicated that most JAG1 was sequestered in the Golgi rather than the pericentrion. In contrast, histological, biochemical, and secretion/trafficking studies indicated that most JAG2 was secreted as a full-length protein, and CHIR and XAV studies suggested that serine/threonine phosphorylation regulated JAG2 stability and/or routing to the plasma membrane (57, 66).

Collectively, these studies identify a complex set of posttranslational modifications that differentially regulate JAG1 and JAG2 location and indicate that Notch signaling in the tracheobronchial epithelium is governed by the proteome as well as the transcriptome. Further analysis of JAG trafficking has the potential to identify treatments that could normalize goblet and ciliated cell frequency in people who have chronic mucosecretory lung diseases and those whose ciliated cell population is depleted by exposure to environmental agents.

A previous study reported that NOTCH1 trafficking to the cell surface was inhibited by GSK3-mediated phosphorylation (67), while the present study showed that GSK3 inhibition resulted in JAG2 degradation. Consequently, GSK3 activity might determine if a cell traffics JAG2 or NOTCH1 to the cell surface. Although the WNT/β-catenin pathway was a likely regulator of GSK3 activity, our previous report found that CHIR and XAV (36) inhibited secretory progenitor commitment and ciliated cell progenitor specification. The present study indicates that both CHIR and XAV antagonized WNT/β-catenin signaling and that the main effect of these drugs was downregulation of Notch signaling. Although these data suggest that Notch signaling is dependent on CTNNB1, a role for WNT signaling was discounted by studies that inhibited WNT secretion or WNT interaction with LRP6/5. A similar WNT ligand-independent role for GSK3 in cell fate specification was previously reported for iPSCs that were undergoing directed differentiation (68) and suggested that establishment of tracheobronchial ligand– and receptor-expressing cells was regulated by a GSK3-dependent and WNT-independent mechanism.

### Conclusion.

This study demonstrates that JAG1 and JAG2 are necessary for cell fate specification within the tracheobronchial epithelium. Differences in JAG1 and JAG2 trafficking raise the possibility that distinct assemblies of these ligands generate Notch signaling environments within the differentiating epithelium.

## Methods

Further information can be found in Supplemental Methods.

### Human TSC recovery, expansion, and ALI culture.

Human bronchial basal cells were recovered as previously described (69, 70) and differentiated using previously reported methods (70). Initial studies used 3 culture media: Wu (71), H&H (36), and complete PneumaCult (Stem Cell Technology).

### ALI culture treatment protocol.

Information regarding specific treatment, analysis day, and concentration of agonists/antagonists is presented in Results and figure legends. Small-molecule agonists and antagonists were purchased from Tocris and dissolved in DMSO at 1000 times the treatment concentration. Vehicle controls were treated with an equal volume of DMSO. Jag1 peptide (AnaSpec, 188-204) was dissolved in 1× PBS at 500 times the treatment concentration. Vehicle controls were treated with an equal volume of PBS. Neutralizing antibodies specific for Jag1 (anti-JAG1.b70) and Jag2 (anti-JAG2.b33) were provided by Genentech (29) and were dissolved in PBS at 100 times the treatment concentration. Vehicle controls were treated with an equivalent volume of PBS.

### Immunofluorescence analysis and quantification.

Differentiation was quantified in ALI cultures using previously reported methods and antibodies (70). Ciliated and goblet cell differentiation intermediates were defined as previously reported (36) and are illustrated in (Supplemental Figure 2).Three individuals, who were blind to the groups, used guide images to classify ciliated and goblet cell differentiation intermediates, and the 3 values were averaged. For analysis of cell surface markers, cultures were fixed with 10% neutral buffered formalin and blocked with 5% bovine serum albumin/1× PBS. Additional antibodies used in this study were as follows: mouse anti-JAG1 C-terminal–specific antibody (BD Biosciences, 612346, 1:50); rabbit anti-JAG2 N-terminal–specific antibody (Cell Signaling Technology [CST], C23D2, 1:50); rabbit anti-JAG2 C-terminal–specific antibody (CST, C83A8, 1:50); goat anti-HES1 (Santa Cruz Biotechnology, sc-13842, 1:40); rabbit anti-RBPJ (MilliporeSigma, AB5790, 1:100). Primary antibodies were detected with Alexa Fluor 488– or Alexa Fluor 594–labeled secondary antibodies (Jackson ImmunoResearch, 1:500). Nuclei were detected with DAPI.

### Cell fractionation.

Cells were separated into cytoplasmic and nuclear fractions using a NE-PER kit (Thermo Fisher Scientific [TFS], P178833). 4× Laemmli’s buffer/10% β-mercaptoethanol (BME) was diluted 1:4 into the protein fraction, and the mixture was incubated at 96°C for 10 minutes.

### Cell lysis.

RIPA buffer (72) was used to lyse cells for immunoprecipitation, polyubiquitination, and Western blot analysis. The apical and basal surfaces of ALI cultures were washed with Hams/F12 at 4°C for 10 minutes. The wash solution was aspirated, and 270 μL RIPA buffer was added to the apical surface. The cell layer was disrupted with a p200 pipette tip and incubated for 20 minutes at 4°C. The lysate was collected in a 1 mL microcentrifuge tube, sonicated 5 times for 30 seconds each time in a Bioruptor (Diagenode) at 4°C, and centrifuged at 11,176*g* for 10 minutes at 4°C. The supernatant was stored at –80°C.

### Immunoprecipitation.

Immunoprecipitation was performed using a modified version of the Pierce Protein Magnetic Bead protocol (TFS, 88848). For each sample, beads (125 μL) were prewashed 3 times with RIPA buffer and resuspended in RIPA buffer with proteinase (Roche, 11836153001) and phosphatase inhibitors (Roche, 04906837001). Washed beads (25 μL) were added to 400–600 μg protein per sample, and the lysate was cleared by rocking for 1 hour at 4°C. The cleared lysate was incubated with a titration-determined amount of antibody (usually 1:100). This mixture was rocked for 1 hour at 4°C. Washed beads (100 μL) were added to the sample and rocked for 2 hours at 4°C. The beads were collected using a neodymium magnet, washed 3 times with RIPA buffer, resuspended in 1× Laemmli’s buffer plus 2.5% BME, and incubated at 96°C for 10 minutes. A portion of the supernatant was kept as the unbound fraction.

### Cell surface biotinylation.

A modified version of the Pierce Cell Surface Protein Biotinylation and Isolation Kit (TFS, A44390) protocol was used to isolate surface proteins. The recommended biotinylation incubation time was increased to 45 minutes, and incubations were done on ice.

### Polyubiquitination.

Polyubiquitinated proteins were isolated using a LifeSensors TUBEs Kit (LifeSensors, UM411M) and a modified protocol. The cell lysate was incubated with 100 μL washed, unconjugated beads at 4°C on a rotating platform for 30 minutes. A bead titration study demonstrated an optimal ratio of 100 μL TUBE-conjugated beads per 1–2 mg protein in a total of 1 mL lysis buffer. The cleared lysate and washed TUBE beads were incubated at 4°C on a rotating platform for 30 minutes. The TUBE beads were collected using a magnet, washed 3 times with PBS/Tween20, and divided into 2 aliquots. One aliquot was treated with buffer, and the other aliquot was treated with 2 μL of 10 μM broad-spectrum deubiquitinase for 2 hours at 30°C with rocking. The beads were resuspended in 1× Laemmli’s buffer/2.5% BME and incubated at 96°C for 10 minutes.

### Western blot analysis and quantification.

Protein concentration, gel electrophoresis and transfer, and blotting were done as previously reported (72). In some instances, blots were stripped by incubation with 1× Western Reprobe (G-Biosciences, 786-119) for 45 minutes at 55°C followed by 3 washes with Tris-buffered saline with TWEEN-20 (CST, 9997) at room temperature. Antibodies used for Western blot studies were as follows: JAG2-N-terminal (CST, C23D2, 1:1000); JAG2-C-terminal (CST, C83A8, 1:1000); GSK3 (MilliporeSigma, 05-412, 1:2000); pGSK3 (MilliporeSigma, 0-413, 1:1000); TOP2A (Abcam, 109524, 1:10000); ATP1A1 (CST, 3010S, 1:2500); JAG1-C-terminal (BD, 612346, 1:1000); TUBA1A (MilliporeSigma, T6793, 1:2000); N-terminal β-catenin (CTNNB1, CST, 9581X, 1:1000); and C-terminal CTNNB1 (BD, 610154, 1:2000).

### RNA preparation and RNA-Seq analysis.

RNA was purified using a RNeasy Kit (Qiagen, 74106) according to the manufacturer’s directions. RNA-Seq and analysis were conducted by the Institute for Genomic Medicine at Nationwide Children’s Hospital as previously reported (73). The data sets generated in this study are available from the corresponding author upon request.

### scRNA-Seq analysis.

Single cells were recovered from ALI cultures using 0.25% Trypsin/2.21 mM EDTA (Corning, 25-053-Cl), and Chromium 10× Genomics single-cell libraries were generated by the Genomics Shared Resource at The Ohio State University Comprehensive Cancer Center. RNA-Seq was done as indicated above. The raw reads were processed with Cell Ranger software 6.0.0 (10× Genomics) using the hg38 transcriptome reference from the GENCODE 25 annotation, and a digital expression matrix was generated with cell barcodes as rows and gene identities as columns. For all data, quality control and filtering were performed to remove cells with a low number of expressed genes (threshold, *n* ≥ 200) and elevated expression of apoptotic transcripts (threshold mitochondrial genes, <25%). Only genes detected in at least 3 cells were included. The expression profiles of cells from the DMSO- and CHIR-treated groups were integrated separately before being clustered using the R software package of Seurat, version 4.0.3 (74). Briefly, the data set was normalized using the SCTransform workflow. Data were normalized, scaled, and integrated using the SCTransform command. Principal component analysis (PCA) was run with 3000 of the most variable genes as input using the RunPCA command. Clusters of similar cells were detected using the Louvain method and visualized with Uniform Manifold Approximation and Projection. A model-based analysis of single-cell transcriptomics framework (75) was used to identify differentially expressed genes from each cluster, and canonical markers of airway cells from previously published single-cell atlases were used for annotation (13, 33, 76). Trajectory inference was performed using Monocle 3 package, version 4.0.3 (61). Briefly, a set of “landmark” cells was selected using kmeans() clustering algorithm in R, and a principal graph that defined the trajectories was identified within the landmark cells. The principal graph was used as a guide to construct a graph of all cells where the pseudotime of each cell was then computed back to assigned root node. Genes that varied in expression over a developmental trajectory were identified using the graphtest() function. Plots were generated within Seurat (https://satijalab.org/seurat/) and Monocle 3 (https://cole-trapnell-lab.github.io/monocle3/) or using the ggplot2 package (https://cran.r-project.org/web/packages/ggplot2/index.html). The data sets generated in this study are available from the corresponding author upon request.

### Statistics.

Experiments were repeated at least 3 times. Statistical analyses were performed using Graph Pad Prism. Outliers were identified using the Rout test. Data normality was evaluated by the Shapiro-Wilk test, the Anderson-Darling test, and/or the D’Agostino-Pearson test. For normally distributed data sets, differences were evaluated using 2-tailed Student’s *t* test. For nonnormally distributed data sets, differences were evaluated by the Mann-Whitney test. Sample size is indicated in the figure legends. *P* values of less than 0.05 were considered significant.

### Study approval.

The Nationwide Children’s Hospital Institutional Review Board approved the human studies. Written informed consent to conduct cell biological studies (e.g., airway epithelial cell isolation, amplification, and differentiation) was obtained from every participant. Each donor gave consent to participate in genomic studies, but they did not give explicit consent to data being posted on a public database. The data sets generated in this study are available from the corresponding author upon request.

## Author contributions

SDR conceived and design the study, acquired laboratory data, interpreted data, and prepared and reviewed the manuscript. CLH, AA, and SWL acquired laboratory data and reviewed the manuscript. SW analyzed RNA-Seq and scRNA-Seq data and reviewed the manuscript. ZHT analyzed scRNA-Seq data and reviewed the manuscript. TC and ECB reviewed and interpreted data and reviewed the manuscript.

## Supplementary Material

Supplemental data

## Version 1 07/12/2022

In-Press Preview

## Version 2 08/08/2022

Electronic publication
