# Supplementary material for: Assemblies of JAG1 and JAG2 determine tracheobronchial cell fate in mucosecretory lung disease
Source: JCI Insight. 2022 Aug 8;7(15):e157380. doi: 10.1172/jci.insight.157380 (PMC9462471; doi:10.1172/jci.insight.157380)
Supplement: Supplemental data [file jciinsight-7-157380-s167.pdf]

SUPPLEMENTAL METHODS

Differentiation model development: Analysis of JAG function required a model system in which changes in cell frequency could be detected. Consequently, we evaluated three media that are commonly used during the differentiation phase of ALI culture, Wu (1), Half&Half (H&H, (2)), and complete Pneumacult (cPC, Stem Cell Technology, Vancouver, BC, Canada). All three media supported epithelial polarization and quantification demonstrated that cell density did not vary as a function of medium or time (SFigure 1A). Mature ciliated and goblet cells were identified by immunostaining for ACT and MUC5B (SFigure 2) and quantification demonstrated that all 3 media supported an increase in ciliated cell frequency between differentiation day (D) 7 and D14 (SFigure 1B). Although, frequency of mature goblet cells did not vary as a function of time in Wu or cPC medium, goblet cell frequency increased significantly in H&H medium (SFig 1C). All 3 media produced well-differentiated epithelial on D21 (SFigure 3A-D).

Based on previous demonstration that Notch signaling regulated ciliated and secretory differentiation (reviewed in (3, 4)), expression of NOTCH receptors was evaluated by western blot. The active (furin cleaved) forms of NOTCH1, 2, and 3 were detected (SFigure 4A-C). Since these studies showed that H&H medium supported an increase in ciliated and goblet cell frequency between D7-D14, generated similar numbers of ciliated and goblet cells on D14, and that the cells expressed furin-activated NOTCH1, 2, and 3, the H&H model was used to evaluate the signaling mechanisms that regulate production of ciliated and goblet cells.

Identification of ciliated cell differentiation intermediates: Previous work demonstrated that ciliated cell differentiation involved a set of intermediate phenotypes (reviewed in (5)). In Stage I, cells exited the cell cycle and expressed a primary cilium (SFigure 2A-B). During Stages II and III, new centrioles were generated and docked at the apical cell surface. Cells that contained many basal bodies and occasional short cilia were termed bristle cells (SFigure 2C-D). Finally, during Stage IV, each docked centriole (now termed a basal body) nucleated a motile 9+2 ciliary

axoneme and the ciliary appendage was formed. Cells defined many long motile cilia were termed ciliated cells, SFigure 2E-F).

Identification of goblet cell differentiation intermediates: Goblet cells also exhibited three morphological phenotypes that were defined by the amount and localization of MUC5B. “Low” cells contained small amounts of MUC5B that was limited to punctate granules (SFigure 2G-H). “Medium” cells contained moderate amounts of MUC5B that was in many well-defined secretory granules (SFigure 2I-J). “High” cells were filled with MUC5B and did not have distinct secretory granules (SFigure 2K-L).

SUPPLEMENTAL FIGURE LEGENDS

Supplemental Figure 1

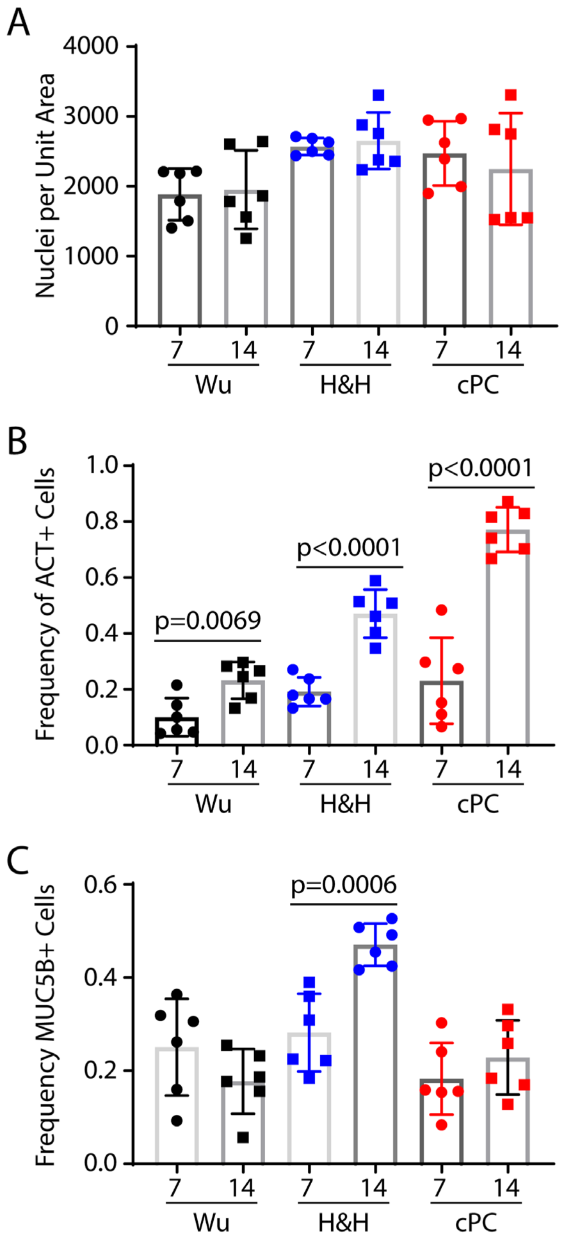

Supplemental Figure 1: Ciliated and goblet cell differentiation as a function of medium type. Human bronchial basal cells were differentiated in ALI cultures using three media: Wu, H&H, and complete Pneumacult (cPC). Cultures were fixed on D7 and D14. **A.** Cell density was determined by quantifying the number of DAPI-stained nuclei per unit area at 200x

magnification. **B.** Ciliated cells were identified by acetylated tubulin (ACT) staining and their frequency was reported as the number ACT+ cells/number nuclei. **C.** Goblet cells were identified by MUC5B staining and their frequency was reported as the number MUC5B+ cells/number nuclei. All data are presented as the Mean ± SD, N=6. Normally distributed data were analyzed by t-test. Non-normally distributed data were analyzed by Mann-Whitney test.

Supplemental Figure 2

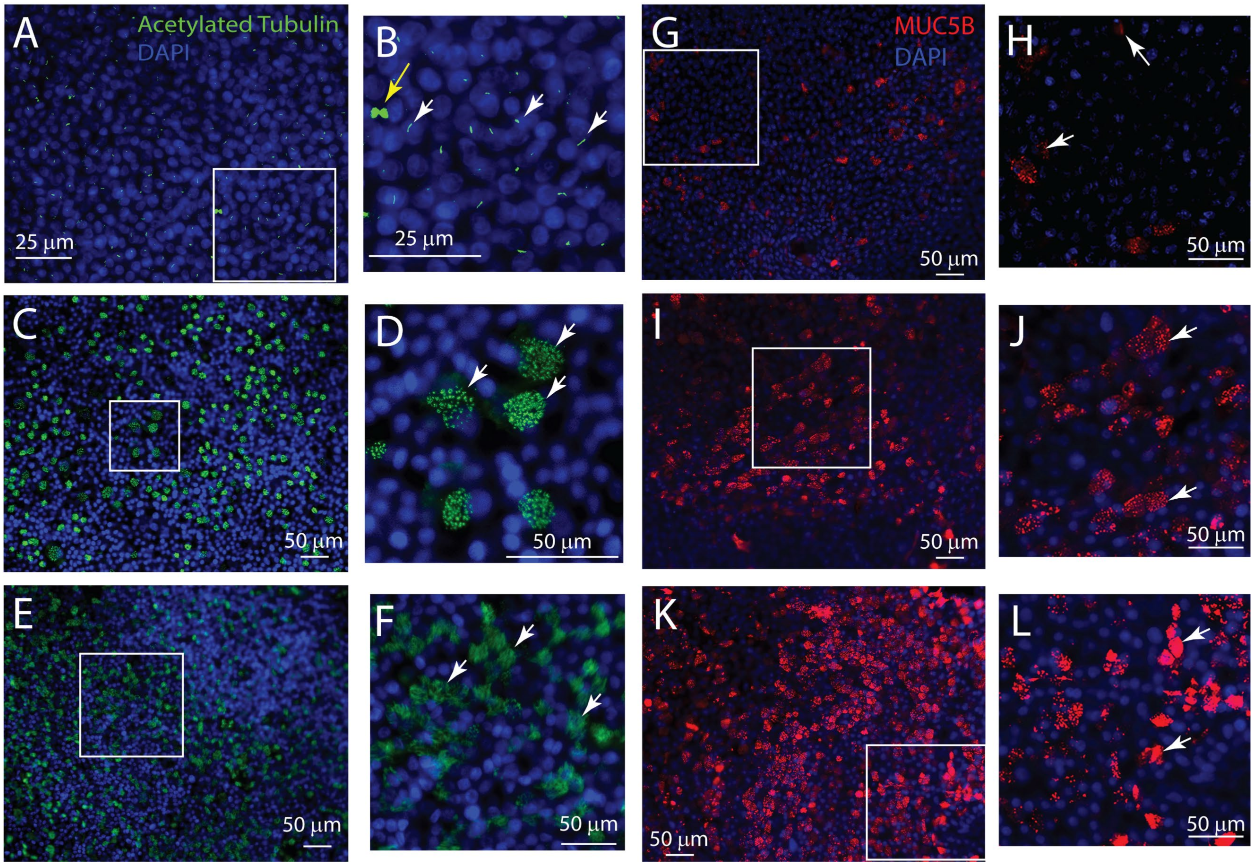

Supplemental Figure 2: Intermediate ciliated and goblet cell phenotypes. Human bronchial basal cells were differentiated in ALI cultures using H&H medium. Scale bars are defined in each panel. **A-B.** Cells defined by a primary cilium were identified by ACT staining on D4. Panel B is a high magnification image of the region identified in panel A. Arrows: white, primary cilium; yellow, mitotic figure. **C-D.** Cells with bristle morphology were identified by ACT staining on D8. Panel D is a high magnification image of the region identified in panel C. Arrows: bristle cells. **E-F.** Ciliated cells defined were identified by ACT staining on D12. Panel F is a high magnification image of the region identified in panel E. Arrows: Ciliated cells. **G-H.** Goblet cells were identified as MUC5B-low on D4. Panel H is a high magnification image of the region identified in panel G. Arrows indicate MUC5B-low cells. **I-J.** Goblet cells identified as MUC5B-medium on D8. Panel J is a high magnification image of the region identified in panel I. Arrows

indicate MUC5B-medium cells. **K-L.** Goblet cells identified as MUC5B-high on D12. Panel L is a high magnification image of the region identified in panel K. Arrows indicate MUC5B-high cells.

Supplemental Figure 3

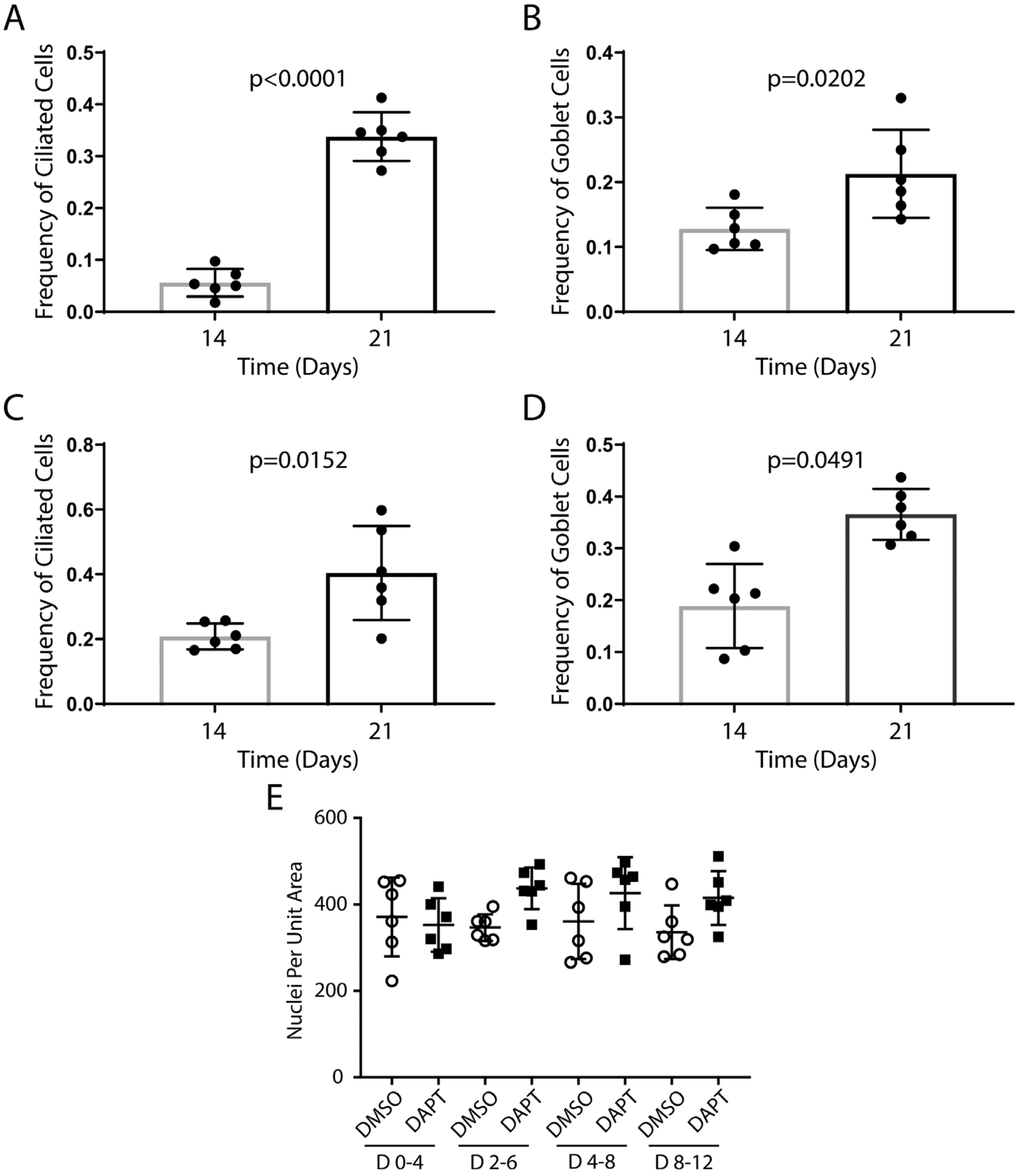

Supplemental Figure 3: Differentiation controls. **A-D.** Cells from two donors (B-C or D-E) were used to generate ALI cultures and differentiated to days 14 or 21 in H&H medium. Ciliated (**A**, **C**) and goblet cell (**B**, **D**) differentiation were quantified. An analysis of a third donor was previously published (2). **E.** Cell density in vehicle and DAPT treated ALI cultures. Human bronchial basal cells were differentiated in ALI cultures using H&H medium. Cells were treated with vehicle (DMSO) or 25 µM DAPT as follows: treatment on D0 and D2 and fixation on D4,

treatment on D2 and D4 and fixation on D6, treatment on D4 and D6 and fixation on D8, or treatment on D8 and D10 and fixation on D12. Nuclei were stained with DAPI and quantified at 400x magnification. All data are presented as the Mean ± SD, N=6. Normally distributed data were analyzed by t-test. Non-normally distributed data were analyzed by Mann-Whitney test.

Supplemental Figure 4

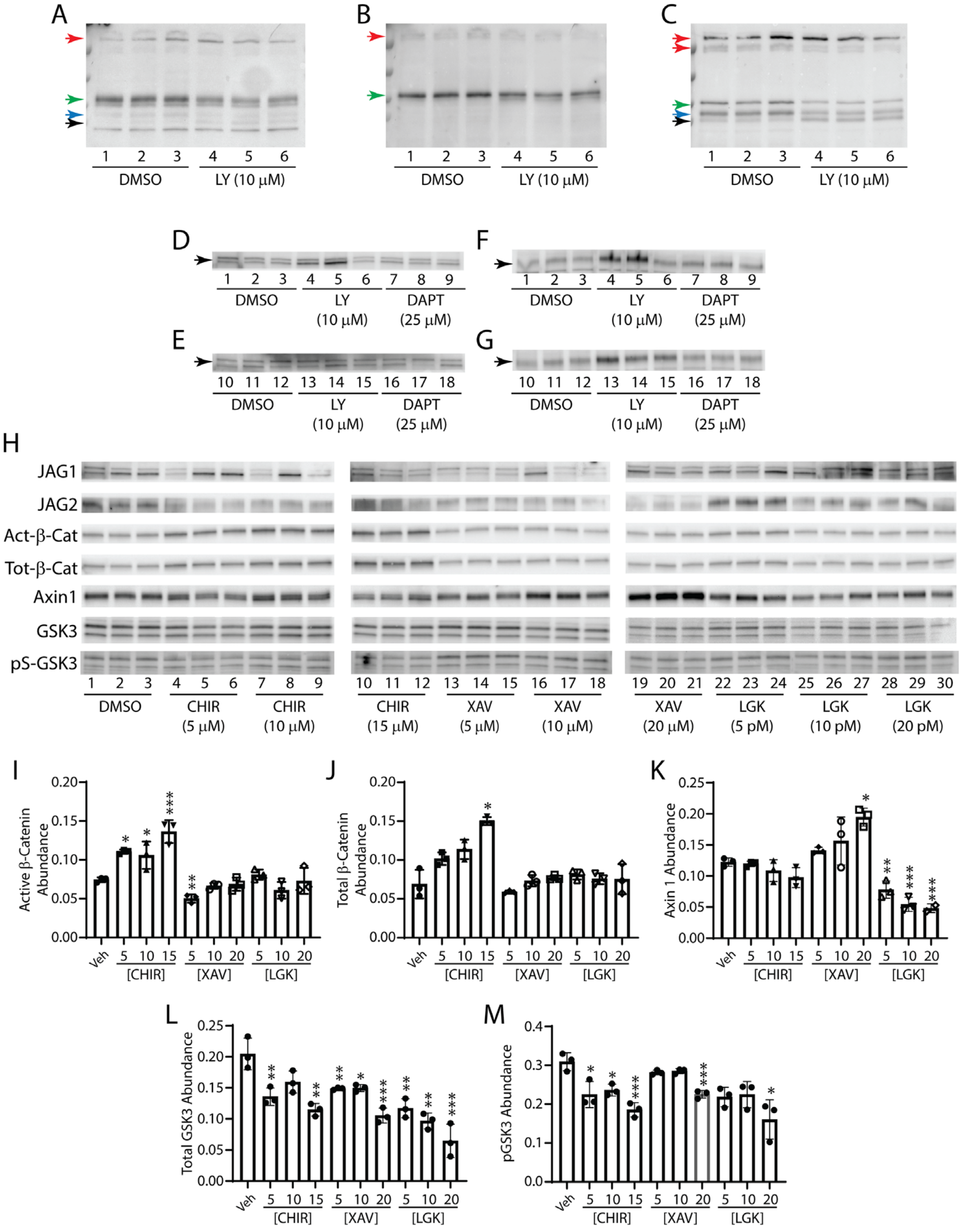

Supplemental Figure 4: Western blots used for quantification of protein abundance. Human bronchial basal cells were differentiated in ALI cultures using H&H medium. Unless otherwise indicated, cultures were treated on D4 and D6 and lysed on D8. **A-C**. Analysis of Notch receptors in cultures that were treated with vehicle (DMSO) or 10 μM LY. NOTCH1 (**A**), NOTCH2 (**B**), and NOTCH3 (**C**). Arrows: red-full length protein; green-furin cleaved protein; blue-protein that has undergone the S2 cleavage by ADAM10 or ADAM17, black-intracellular

domain peptide. **D-E.** Analysis of full-length JAG1 in cultures that were treated with vehicle (DMSO), 10 μM LY, or 25 μM DAPT on D4 and D6 and lysed on D8 (**D**) or treated on D8 and D10 and lysed on D12 (**E**). **F-G.** Analysis of full-length JAG2 in cultures that were treated with vehicle (DMSO), 10 μM LY, or 25 μM DAPT on D4 and D6 and lysed on D8 (**F**) or treated on D8 and D10 and lysed on D12 (**G**). **H.** Analysis of various proteins in cultures that were treated with vehicle (DMSO), CHIR, XAV, or LGK. Full-length proteins were analyzed. **I-M.** Quantification of active CTNNB1 (**I**), total CTNNB1 (**J**), Axin1 (**K**), total GSK3 (**L**), and phospho-S GSK3 (**M**) protein abundance in cultures that were treated with CHIR, XAV, or LGK. CHIR and XAV concentrations are in μM. LGK concentration is in pM. JAG1 and JAG2 quantification is in Fig 5. All data are presented as the Mean ± SD, N=3. Normally distributed data were analyzed by t-test. Non-normally distributed data were analyzed by Mann-Whitney test. Asterisks: * $p<0.05$, ** $p<0.01$, *** $p<0.001$.

Supplemental Figure 5

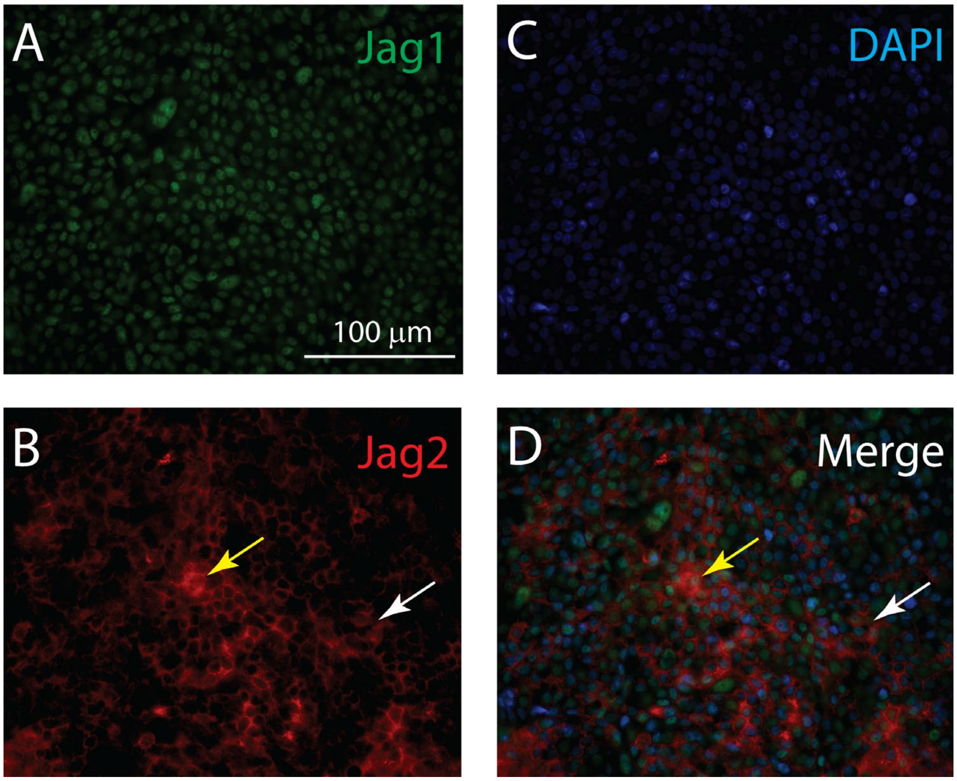

Supplemental Figure 5: JAG1 and JAG2 localization. Human bronchial basal cells were differentiated in ALI cultures using H&H medium. JAG1 (green, **A** and **D**), JAG2 (red, **B** and **D**) and (DAPI (blue, **C** and **D**). Arrows: yellow-high JAG2 expression; white-low JAG2 expression. All images are shown at the same magnification. Scale bar, 100 μm.

Supplemental Figure 6

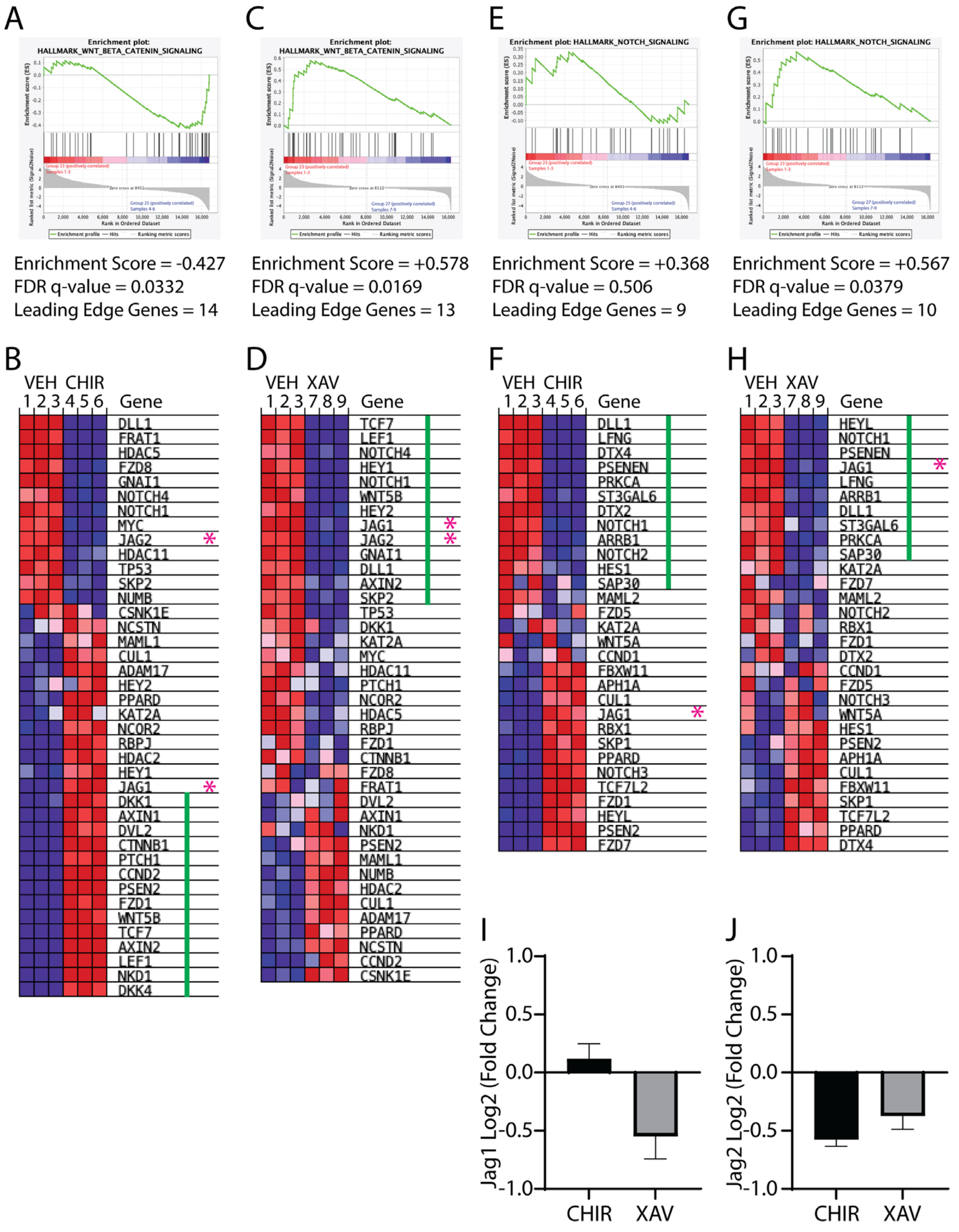

Supplemental Figure 6: Gene expression and gene set enrichment analysis. Human bronchial basal cells were differentiated in ALI cultures using H&H medium. Cultures were treated with vehicle, 10 µM CHIR or 10 µM XAV on D4 and D6 and RNA was purified on D8. Transcriptional changes were interrogated by RNA-sequencing and Gene Set Expression Analysis (GSEA). **A.** GSEA for WNT/β-catenin genes in vehicle and CHIR treated cultures. **B.** Heat map representation of WNT/β-catenin genes in vehicle and CHIR treated cultures. Each column is a

sample: samples 1-3 vehicle treated (VEH), samples 4-6 CHIR treated. Each row is a gene. Red indicates upregulation. Blue indicates downregulation. Leading-edge genes are indicated by the green line. Jag1 and Jag2 are indicated by the asterisks. **C.** GSEA for WNT/β-catenin genes in vehicle and XAV treated cultures. **D.** Heat map representation of WNT/β-catenin genes in vehicle and XAV treated cultures. Each column is a sample: samples 1-3 vehicle (VEH treated), samples 7-9 XAV treated. Each row is a gene. Red indicates upregulation. Blue indicates downregulation. Leading-edge genes are indicated by the green line. Jag1 and Jag2 are indicated by the asterisks. **E.** GSEA for Notch genes in vehicle and CHIR treated cultures. **F.** Heat map representation of Notch genes in vehicle and CHIR treated cultures. Each column is a sample: samples 1-3 vehicle (VEH treated), samples 4-6 CHIR treated. Each row is a gene. Red indicates upregulation. Blue indicates downregulation. Leading-edge genes are indicated by the green line. Jag1 and Jag2 are indicated by the asterisks. **G.** GSEA for Notch genes in vehicle and XAV treated cultures. **H.** Heat map representation of Notch genes in vehicle and XAV treated cultures. Each column is a sample: samples 1-3 vehicle (VEH treated), samples 7-9 XAV treated. Each row is a gene. Red indicates upregulation. Blue indicates downregulation. Leading-edge genes are indicated by the green line. *Jag1* and *Jag2* are indicated by the asterisks. **I.** Analysis of *Jag1* gene expression in vehicle, CHIR, and XAV treated cultures. Data are presented as the Log2(fold change). The Mean ± SD (n=3). Data were analyzed by the Mann-Whitney test. No significant differences were detected. **J.** Analysis of *Jag2* gene expression in vehicle, CHIR, and XAV treated cultures. Data are presented as the Log2(fold change). All data are presented as the Mean ± SD (n=3). Normally distributed data were analyzed by t-test. Non-normally distributed data were analyzed by Mann-Whitney test. No significant differences were detected.

Supplemental Figure 7

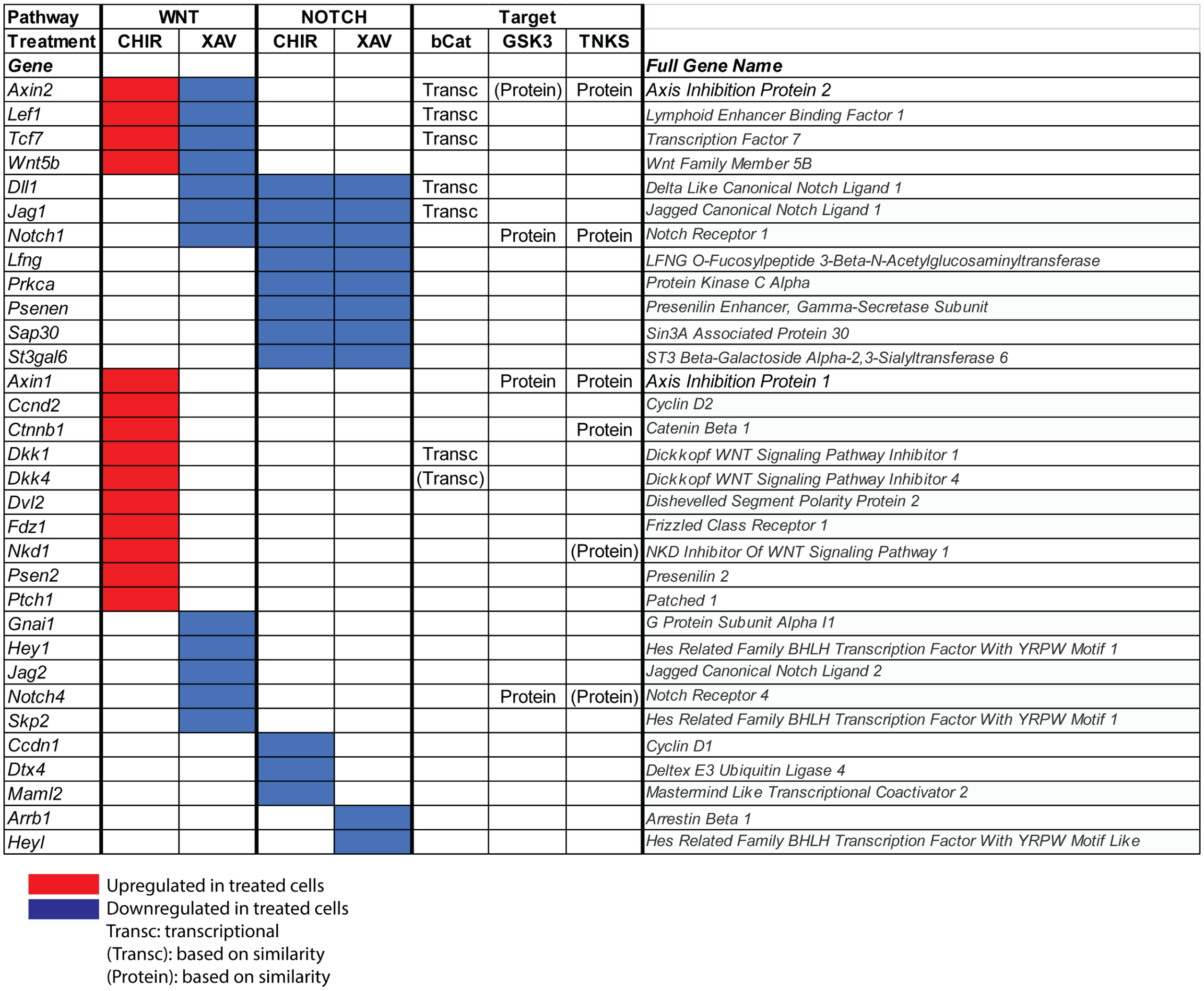

Pathway	WNT		NOTCH		Target			
Treatment	CHIR	XAV	CHIR	XAV	bCat	GSK3	TNKS	
Gene								***Full Gene Name***
Axin2					Transc	(Protein)	Protein	*Axis Inhibition Protein 2*
Lef1					Transc			*Lymphoid Enhancer Binding Factor 1*
Tcf7					Transc			*Transcription Factor 7*
Wnt5b								*Wnt Family Member 5B*
Dll1					Transc			*Delta Like Canonical Notch Ligand 1*
Jag1					Transc			*Jagged Canonical Notch Ligand 1*
Notch1						Protein	Protein	*Notch Receptor 1*
Lfng								*LFNG O-Fucosylpeptide 3-Beta-N-Acetylglucosaminyltransferase*
Prkca								*Protein Kinase C Alpha*
Psenen								*Presenilin Enhancer, Gamma-Secretase Subunit*
Sap30								*Sin3A Associated Protein 30*
St3gal6								*ST3 Beta-Galactoside Alpha-2,3-Sialyltransferase 6*
Axin1						Protein	Protein	*Axis Inhibition Protein 1*
Ccnd2								*Cyclin D2*
Ctnnb1							Protein	*Catenin Beta 1*
Dkk1					Transc			*Dickkopf WNT Signaling Pathway Inhibitor 1*
Dkk4					(Transc)			*Dickkopf WNT Signaling Pathway Inhibitor 4*
Dvl2								*Dishevelled Segment Polarity Protein 2*
Fdz1								*Frizzled Class Receptor 1*
Nkd1							(Protein)	*NKD Inhibitor Of WNT Signaling Pathway 1*
Psen2								*Presenilin 2*
Ptch1								*Patched 1*
Gnai1								*G Protein Subunit Alpha I1*
Hey1								*Hes Related Family BHLH Transcription Factor With YRPW Motif 1*
Jag2								*Jagged Canonical Notch Ligand 2*
Notch4						Protein	(Protein)	*Notch Receptor 4*
Skp2								*Hes Related Family BHLH Transcription Factor With YRPW Motif 1*
Ccdn1								*Cyclin D1*
Dtx4								*Deltex E3 Ubiquitin Ligase 4*
Maml2								*Mastermind Like Transcriptional Coactivator 2*
Arrb1								*Arrestin Beta 1*
Heyl								*Hes Related Family BHLH Transcription Factor With YRPW Motif Like*

Supplemental Figure 7: Analysis of leading-edge genes. ALI cultures were treated with vehicle, 10 μM CHIR, or 10 μM XAV on D4 and D6 and RNA was purified on D8. Transcriptional changes were interrogated by RNA-sequencing and Gene Set Expression Analysis (GSEA). Leading-edge genes, which determine the gene set Enrichment Score, were extracted and organized to indicate similarities/differences across the treatments and gene sets. Red indicates downregulation. Blue indicates upregulation. Transc: Transcriptional targets of WNT/β-catenin were based on literature reports. (transc): WNT/β-catenin targets identified by similarity. Protein: Targets of glycogen synthase kinase (GSK3) and tankyrase (TNKS) were based on literature reports. (protein): GSK3 and TNKS targets identified by similarity.

Supplemental Figure 8

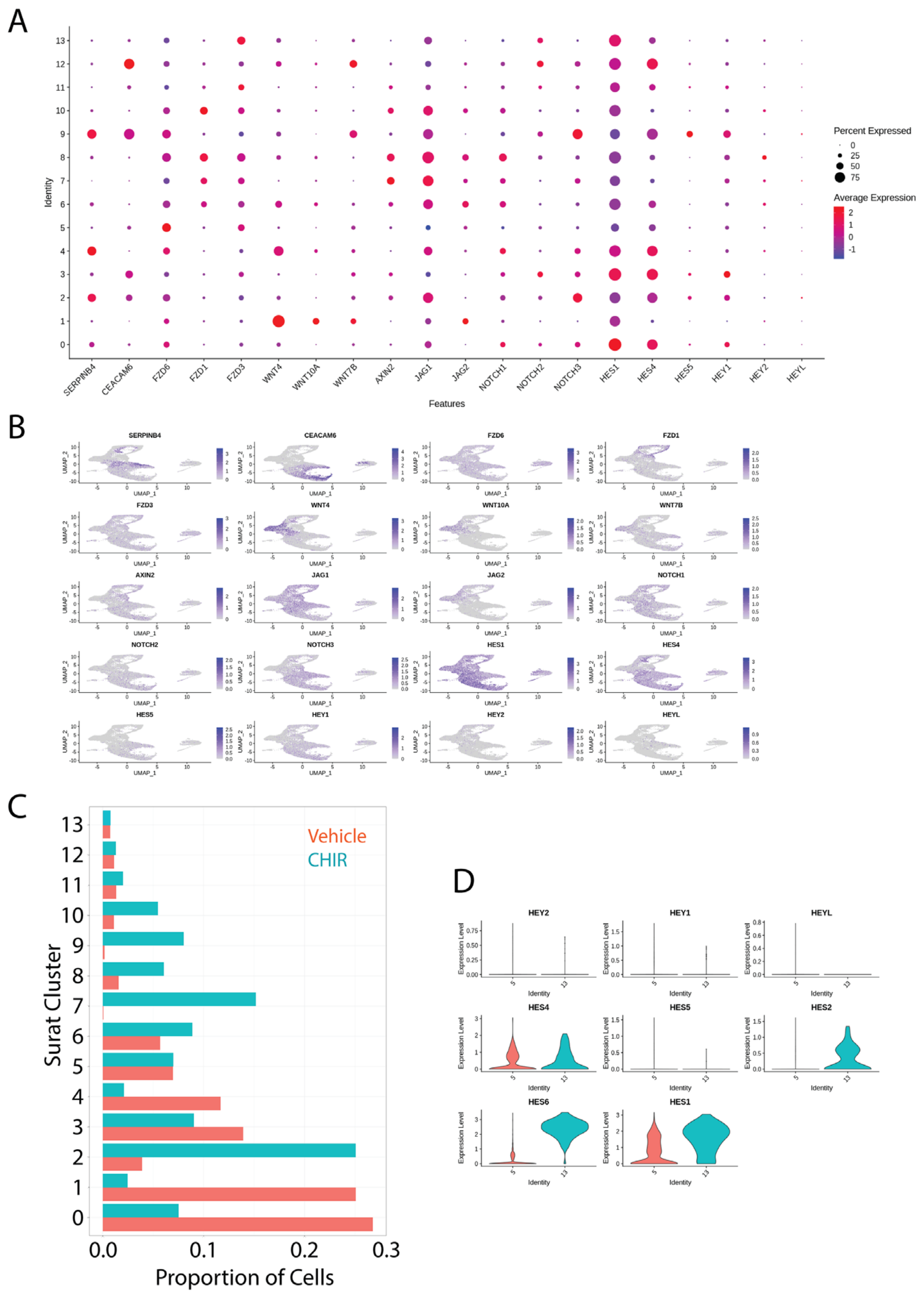

Supplemental Fig 8: Single cell RNAseq analysis. Human bronchial basal cells were differentiated in ALI cultures using H&H medium, treated with vehicle (DMSO) or 10 μM CHIR on D4 and D6, and harvested for scRNAseq analysis on D8. **A.** Dot plot of marker genes (*Serpin4* and *Ceacam6*), Frizzled receptors (*Fzd6*, 1, 3), WNT ligands (*Wnt4*, 10A, 7B), WNT/β-catenin target (*Axin2*), Notch ligands (*Jag1*, 2), Notch receptors (*Notch1*, 2, 3), and Notch targets (*Hes1*, 4, and 5 and *Hey1*, 2, and L). Pathway analysis is limited to differentially

expressed genes within the WNT/β-catenin and Notch pathways. **B.** Cluster analysis of marker mRNA (*Serpinb4* and *Ceacam6*), Frizzled receptors (*Fxd6, 1, 3*), WNT ligands (*Wnt4, 10A, 7B*), WNT/β-catenin target (*Axin2*), Notch ligands (*Jag1, 2*), Notch receptors (Notch1, 2, 3), and Notch targets (Hes1, 4, and 5 and Hey1, 2, and L). Pathway analysis is limited to differentially expressed genes within the WNT/β-catenin and Notch pathways. **C.** Normalized number of cells per Surat Cluster. **D.** Violin plots of normalized expression of for *Hey* and *Hes* family genes in the ciliated lineage (clusters 5 and 13).

STable 1: Gene expression as a function of cluster.

References

1. Wu R. Culture of normal human airway epithelial cells and measurement of mucin synthesis and secretion. Methods in molecular medicine. 2000;44:31-9.
2. Malleske DT, Hayes D, Jr., Lallier SW, Hill CL, Reynolds SD. Regulation of human airway epithelial tissue stem cell differentiation by beta-catenin, P300, and CBP. Stem Cells. 2018.
3. Carraro G, Stripp BR. A new notch for lung stem cells. Cell stem cell. 2015;16(2):107-9.
4. Kiyokawa H, Morimoto M. Notch signaling in the mammalian respiratory system, specifically the trachea and lungs, in development, homeostasis, regeneration, and disease. Dev Growth Differ. 2020;62(1):67-79.
5. Boutin C, Kodjabachian L. Biology of multiciliated cells. Current opinion in genetics & development. 2019;56:1-7.
